# Interfacial Electronic Interactions Between Ultrathin NiFe‐MOF Nanosheets and Ir Nanoparticles Heterojunctions Leading to Efficient Overall Water Splitting

**DOI:** 10.1002/advs.202401780

**Published:** 2024-04-26

**Authors:** Cong Li, Wei Zhang, Yongyong Cao, Jun‐Yang Ji, Zhao‐Chen Li, Xu Han, Hongwei Gu, Pierre Braunstein, Jian‐Ping Lang

**Affiliations:** ^1^ College of Chemistry Chemical Engineering and Materials Science Soochow University Suzhou Jiangsu 215123 P. R. China; ^2^ State Key Laboratory of Organometallic Chemistry Shanghai Institute of Organic Chemistry Chinese Academy of Sciences Shanghai 200032 P. R. China; ^3^ College of Biological Chemical Science and Engineering Jiaxing University Jiaxing Zhejiang 314001 P. R. China; ^4^ Université de Strasbourg – CNRS Institut de Chimie (UMR 7177 CNRS) 4 rue Blaise Pascal‐CS 90032 Strasbourg 67081 France

**Keywords:** electronic modulation, heterojunctions, Ir nanoparticles, NiFe‐MOF nanosheets, overall water splitting

## Abstract

Creating specific noble metal/metal‐organic framework (MOF) heterojunction nanostructures represents an effective strategy to promote water electrolysis but remains rather challenging. Herein, a heterojunction electrocatalyst is developed by growing Ir nanoparticles on ultrathin NiFe‐MOF nanosheets supported by nickel foam (NF) via a readily accessible solvothermal approach and subsequent redox strategy. Because of the electronic interactions between Ir nanoparticles and NiFe‐MOF nanosheets, the optimized Ir@NiFe‐MOF/NF catalyst exhibits exceptional bifunctional performance for the hydrogen evolution reaction (HER) (*η*_10_ = 15 mV, *η* denotes the overpotential) and oxygen evolution reaction (OER) (*η*_10_ = 213 mV) in 1.0 m KOH solution, superior to commercial and recently reported electrocatalysts. Density functional theory calculations are used to further investigate the electronic interactions between Ir nanoparticles and NiFe‐MOF nanosheets, shedding light on the mechanisms behind the enhanced HER and OER performance. This work details a promising approach for the design and development of efficient electrocatalysts for overall water splitting.

## Graphical abstract

A meticulously designed heterojunction bifunctional catalyst Ir@NiFe‐MOF/NF, derived from anchoring Ir nanoparticles in NiFe‐MOF/NF nanosheet arrays, exhibits a robust Ir─O─Ni/Fe interface interaction confirmed by XPS, Raman, and XAFS analyses. Such interaction augments H_2_O and intermediate adsorption, resulting in the superior HER and OER activities of the catalyst, which can serve as a promising bifunctional candidate for efficient electrocatalytic water splitting.

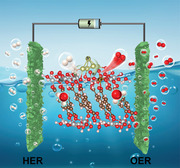

## 1 Introduction

Electrocatalytic water splitting, a promising technology for meeting future energy demands and addressing environmental concerns,^[^ ^1^ ^]^ has faced limitations due to the sluggish kinetics of both the cathode hydrogen evolution reaction (HER) and the anode oxygen evolution reaction (OER).^[^ ^2^ ^]^ Overcoming these challenges necessitates the development of cost‐effective and stable electrocatalysts capable of efficiently and continuously converting H_2_O into high‐purity hydrogen and oxygen.^[^ ^3^ ^]^ However, given the disparities in the catalytic mechanisms governing OER and HER, different catalysts need designed and developed to minimize the overpotential for electrocatalytic water splitting,^[^ ^4^ ^]^ which is undoubtedly complicated and costly. Hence, the continued development of low‐cost, efficient, and stable bifunctional electrocatalytic materials for overall efficient water splitting holds significant promises but also presents ongoing challenges.

Metal‐organic frameworks (MOFs), a class of porous crystalline materials composed of metal centers and organic bridging ligands, have gained popularity in electrocatalysis due to their precise structure, tunability, periodic porous framework, and abundant active sites.^[^ ^5^ ^]^ However, pristine MOFs typically exhibit unsatisfactory electrocatalytic activity for the OER and HER.^[^ ^6^ ^]^ To improve their OER performances, many studies have focused on metal doping in MOFs.^[^ ^7^ ^]^ Nevertheless, enhancing their HER activity remains challenging. Noble metals such as Pt, Ir, and Ru are known for their suitable adsorption free energy for ^*^H (^*^H denotes a hydrogen intermediate adsorbed on a catalytic site) and HER activity.^[^ ^8^ ^]^ Therefore, associating MOFs to noble metals through heterojunction engineering could represent an attractive strategy for designing efficient catalysts for overall water splitting, as also suggested by recent works.^[^ ^9^ ^]^ MOFs, with their large specific surface area, unique surface chemistry, and abundant functional groups, serve as ideal precursors for dispersing noble metals and forming noble metal nanoparticle (NP)‐MOF heterojunctions.^[^ ^10^ ^]^ The exposed Lewis base sites on MOF surfaces can strongly interact with noble metal NPs, allowing a fine‐tuning of the coordination environment and electronic structure of the active site.^[^ ^11^ ^]^ This hybridization of noble metals with MOFs has proven to be effective in enhancing catalytic activity. For instance, Sun et al. developed a hybrid catalyst (Ni‐MOF@Pt) with excellent HER activity in both acidic and basic electrolytes by in situ growth of Pt NPs on 2D MOF nanosheets.^[^ ^12^ ^]^ Peng et al. anchored quantum‐sized Ru NPs on Ni‐MOF and observed excellent HER activity across a broad range of pH values.^[^ ^13^ ^]^ The nature of the reducing agents and solvents can also impact the formation of metal NP‐MOF heterojunctions. Huang et al. investigated the reduction kinetics of precious metal ions and observed a strong influence of the solvent on their reduction and the aggregation tendency of the noble metal NPs.^[^ ^14^ ^]^ Furthermore, Peng et al. used Ir doping in NiCo layered double hydroxide (LDH) to achieve high electrocatalytic overall water‐splitting activity.^[^ ^15^ ^]^ Although known as an excellent catalyst for OER and HER, Ir has received limited attention as a heterojunction material with MOFs.^[^ ^16^ ^]^ Therefore, a rational design and development of Ir NP/MOF heterojunction electrocatalysts, along with an in‐depth exploration of the influence of interface electronic regulation on HER and OER activity, are required for improving overall water splitting electrocatalysts.

As part of our continuing interest for this promising but challenging area,^[^ ^17^ ^]^ we have now designed an Ir@NiFe‐MOF heterojunction integrated electrocatalytic electrode by anchoring Ir NPs onto a nickel foam (NF)‐supported ultrathin NiFe‐MOF nanosheet array. This is achieved through a straightforward solvothermal process and subsequent galvanic reduction of the Ir(III) ions. A crucial aspect of this approach is the formation of unique Ir─O─Ni/Fe bonds between the Ir NPs and the NiFe‐MOF, which modulates the interface electronic structure and significantly enhances the electrochemical activity. The optimized Ir@NiFe‐MOF/NF will be shown to exhibit remarkable electrocatalytic activity for both the alkaline HER and OER. Both density functional theory (DFT) calculations and experimental results demonstrate the importance of the strong Ir─O─Ni/Fe interactions at the heterojunction interface in adjusting the electronic structure of the active center and shifting the d‐band center of the catalyst, ultimately resulting in enhanced overall water splitting performance.

## 2 Results and Discussion

### 2.1 Preparation and Characterization of Catalysts

**Scheme 1:**
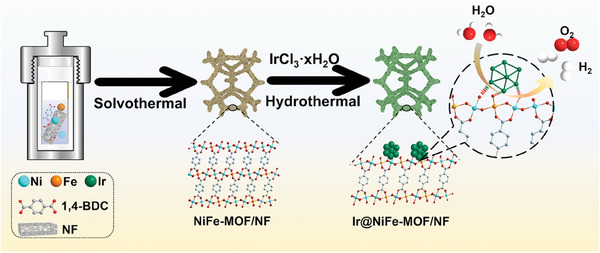
Schematic illustration of the preparation of Ir@NiFe‐MOF/NF (NF = Ni foam; 1,4‐H_2_BDC = terephthalic acid).

**Figure 1:**
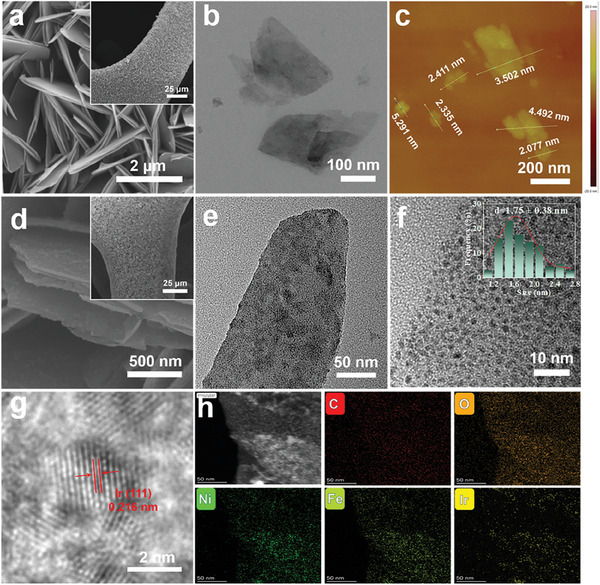
a) SEM, b) TEM, and c) AFM images of NiFe‐MOF/NF. d) SEM, e,f) TEM, and g) HRTEM images of Ir@NiFe‐MOF/NF and h) elemental mappings of C, O, Ni, Fe, and Ir in Ir@NiFe‐MOF/NF.

As depicted in **Scheme** **1**, the Ir@NiFe‐MOF/NF self‐supporting catalytic electrode was successfully prepared through a two‐step process involving a simple solvothermal reaction and subsequent hydrothermal treatment. Initially, ultrathin NiFe‐MOF nanosheet arrays were grown on a nickel foam (NF) using Ni(NO_3_)_2_·6H_2_O and FeCl_3_·6H_2_O as metal sources, and terephthalic acid (1,4‐H_2_BDC) as a ligand in a mixture of N,N‐dimethylformamide (DMF) and H_2_O. The powder X‐ray diffraction (PXRD) patterns of the resulting NiFe‐MOF matched those simulated by single crystal data of Ni‐MOF (CCDC no. 638 866)^[^ ^18^ ^]^ (Figure S1a, Supporting Information). Scanning electron microscopy (SEM) image (**Figure** **1**a) exhibited the uniform and dense growth of NiFe‐MOF nanosheets on the NF substrate, with energy‐dispersive X‐ray spectroscopy (EDS) mapping confirming the uniform distribution of the elements C, O, Ni, and Fe in the MOF nanosheets and the homogeneous Fe doping of ≈0.4 wt.% (Figures S1 and S2, Supporting Information), consistent with the inductively coupled plasma‒optical emission spectrometry (ICP‐OES) result (0.36 wt.%) (Table S1, Supporting Information). Transmission electron microscopy (TEM) and atomic force microscopy (AFM) images revealed the ultrathin nanosheet morphology of NiFe‐MOF with a thickness of ca. 2–4 nm (Figure 1b,c). In order to elucidate the reduction process of Ir^3+^ and the formation of Ir NPs, a series of control experiments involving NF and Ir^3+^ in an aqueous solution were performed. The results confirmed the reduction of Ir^3+^ to Ir NPs in the presence of NF, and the oxidation of partial NF (Ni(0)) into Ni^2+^ ions, which entered into the solution (Figure S3, Supporting Information). We thus concluded that Ir^3+^ ions were reduced by NF (Ni(0)) to form Ir(0), while the resulting oxidized species (Ni^2+^) went into the reaction solution. Subsequently, the generated Ir(0) atoms were further anchored and aggregated on the surface of NiFe‐MOF nanosheets to afford Ir NPs, thereby forming Ir@NiFe‐MOF/NF. Notably, the reduction reaction is thermodynamically favorable,^[^ ^19^ ^]^ given the positive total potential of Ir and Ni^2+^ produced during the reaction of Ir^3+^ and Ni (ΔE = E_Ir_^III^_/Ir_^0^ − E_Ni_^II^_/Ni_^0^ > 0). The galvanic reaction proceeded as follows:

(1) $$Ni^{2+}\;+\;2e^{-}⇌\mathrm{Ni},E_{0}=-0.25V$$

(2) $$Ir^{3+}+3e^{-}⇌\mathrm{Ir},E_{0}=+1.19V$$

(3) $$3\mathrm{Ni}+2Ir^{3+}⇌3Ni^{2+}+2\mathrm{Ir},\Delta E>0$$

The abundance of electron‐rich oxygen atoms on the surface of the NiFe‐MOF nanosheets provides uniform anchoring sites during the reduction of the Ir ion^[^ ^20^ ^]^ (Figure S4, Supporting Information). As evident in the SEM and TEM images (Figure 1d–f), ultrasmall Ir NPs were effectively anchored on the NiFe‐MOF nanosheets, with particle sizes predominantly falling within a narrow range of 1.0–3.0 nm and an average size of 1.75 nm. The ultrathin nanosheet structure of NiFe‐MOF, along with the immobilized oxygen atoms on the surface, not only hindered Ir NPs aggregation but also provided an abundance of electrochemically active sites, contributing to improved HER/OER kinetics^[^ ^21^ ^]^ An interplanar spacing of 0.216 nm, corresponding to the (111) crystal plane of cubic Ir, confirmed the presence of Ir NPs (Figure 1g).^[^ ^22^ ^]^ Moreover, the corresponding high‐resolution EDS mappings demonstrated the even distribution of Ni, Fe, Ir, C, and O elements (Figure 1h), which implies that Ir NPs grow uniformly on the NiFe‐MOF surface.

**Figure 2:**
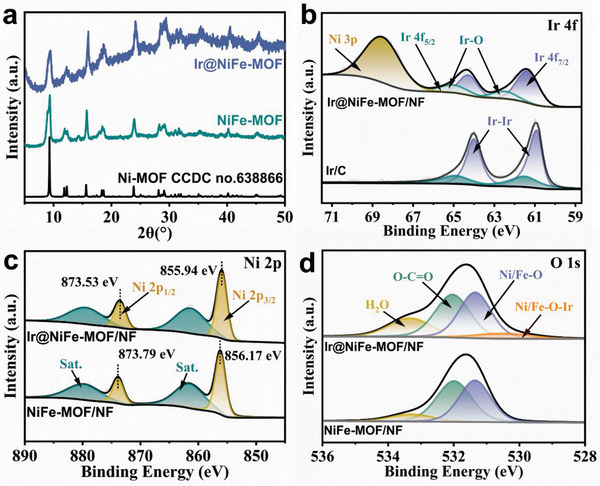
PXRD patterns of NiFe‐MOF/NF and Ir@NiFe‐MOF/NF a), High‐resolution XPS spectra of Ir 4f b), Ni 2p c), and O 1s d) of NiFe‐MOF/NF and Ir@NiFe‐MOF/NF.

The thickness of the Ir@NiFe‐MOF nanosheets grown on NF was observed between 2.9 and 3.3 µm, as determined from cross‐section SEM images (Figure S5, Supporting Information). By measuring the difference in mass after high‐power sonication, the weight of Ir@NiFe‐MOF grown on NF was estimated, resulting in a catalyst loading density of 1.85 ± 0.5 mg·cm^−2^ (Table S2, Supporting Information). EDS mapping confirmed the uniform distribution of Ir throughout Ir@NiFe‐MOF/NF, with an Ir loading of ≈8.2 wt.% (Figure S6, Supporting Information). This was further confirmed by ICP‒OES, which gave the Ir and Fe contents to be 8.14 and 0.35 wt.%, respectively (Table S1, Supporting Information), in agreement with the EDS results. Furthermore, considering that the Fe content of Ir@NiFe‐MOF/NF may affect the electrochemical performance, we optimized the Fe doping level in the NiFe‐MOF/NF precursors. According to the PXRD results, it was evident that increasing Fe dosage from 0 to 0.05 mmol maintained the original structure of NiFe‐MOF, although the PXRD patterns of Fe‐MOF did not match the simulated ones (Figure S7, Supporting Information). SEM images indicated that excessive Fe doping disrupted the morphology of the NiFe‐MOF/NF ultra‐thin nanosheet array, resulting in a disordered block‐like morphology in NiFe_0.5_‐MOF/NF. However, the Fe‐MOF surfaces resembled bare NF, indicating that Fe‐MOF did not grow on the nickel foam (Figure S8, Supporting Information). Nevertheless, Ir NPs can still grow on Ni‐MOF/NF, NiFe_0.1_‐MOF/NF, and NiFe_0.5_‐MOF/NF, with Fe contents being ≈0, 0.2, and 0.6 wt.%, respectively (Figure S9, Supporting Information). Therefore, we considered Ir@NiFe_0.3_‐MOF/NF (or simply Ir@NiFe‐MOF/NF) as the best sample for optimizing the Fe doping content. The PXRD patterns of Ir@NiFe‐MOF/NF closely resembled those of pristine NiFe‐MOF, establishing the preservation of the MOF structure during the generation of the Ir NPs (**Figure** **2**a). Notably, no Ir NP peak was observed owing to the low Ir content. Samples of Ir@NiFe‐MOF/NF with varying Ir loadings also displayed similar PXRD patterns (Figure S10, Supporting Information). When the Ir loading increased, a peak at ≈40.7° attributable to Ir NPs emerged in the PXRD patterns of Ir@NiFe‐MOF/NF‐1000. SEM images and EDS spectra further indicated gradual aggregation of the Ir NPs and increasing loading from 2.8 to 27.9 wt.% when the amount of Ir^3+^ solution was increased (Figure S11, Supporting Information).

X‐ray photoelectron spectroscopy (XPS) was conducted to investigate the surface chemical composition and electronic structure of Ir@NiFe‐MOF/NF. The XPS measurement was performed on the Ir@NiFe‐MOF nanosheets obtained through ultrasonic exfoliation from Ir@NiFe‐MOF/NF, thereby minimizing any influence from the NF substrate. The XPS survey spectra of Ir@NiFe‐MOF/NF confirmed the presence of Ir, Ni, Fe, C, and O (Figure S12a,b, Supporting Information), which is consistent with the EDS results. The Ir 4f high‐resolution scan spectra of Ir@NiFe‐MOF/NF showed two peaks at 60.85 and 63.78 eV (Figure 2b), corresponding to 4f_7/2_ and 4f_5/2_ of metallic Ir,^[^ ^23^ ^]^ respectively. Another two peaks at 62.01 and 64.52 eV were assigned to Ir─O, formed at the edges of the Ir NPs with the surface oxygen of NiFe‐MOF nanosheets.^[^ ^24^ ^]^ Importantly, the Ir 4f peaks of metallic Ir in Ir@NiFe‐MOF/NF were positively shifted by ≈0.4 eV toward higher binding energy compared to the Ir NPs of Ir/C (60.40 eV/63.51 eV), confirming that the Ir NPs on the surface of NiFe‐MOF nanosheets were strongly electronically affected by the Ir─O bonds. The Ni 2p region was deconvoluted into two pairs of peaks (Figure 2c), corresponding to Ni 2p_3/2_ (856 eV) and Ni 2p_1/2_ (874 eV), along with a pair of satellite peaks (denoted as “Sat.”).^[^ ^25^ ^]^ Notably, there was a negative binding energy shift of ≈0.23 eV for the Ni 2p_3/2_ and 2p_1/2_ spin‐splitting peaks in Ir@NiFe‐MOF/NF (located at 855.94 and 873.53 eV) compared to NiFe‐MOF/NF (located at 856.17 and 873.79 eV), indicating that Ir NPs transferred some of their electron density to Ni atoms via Ir─O─Ni bonds. Similar results were observed in the high‐resolution XPS spectrum of Fe 2p (Figure S12c, Supporting Information). Additionally, a new peak centered at 530.5 eV was identified in the O1s XPS spectra of Ir@NiFe‐MOF/NF compared to NiFe‐MOF/NF and was associated with the Ni/Fe─O─Ir bond (Figure 2d).^[^ ^9^ ^]^ And the peaks at 531.36 and 531.33 eV were attributed to the Ni/Fe─O species in NiFe‐MOF/NF and Ir@NiFe‐MOF/NF,^[^ ^26^ ^]^ respectively. The slight negative shift in binding energy for Ir@NiFe‐MOF/NF suggests the charge transfer from Ir NPs to Ni/Fe through Ir─O─Ni/Fe bonding, increasing Ni/Fe charge density and lowering Ni/Fe─O binding energy. This indicates the intricate electronic interactions at the Ir@NiFe‐MOF/NF heterojunction interface, highlighting the impact of Ir─O─Ni/Fe bonding on the electronic structure of the catalyst. Furthermore, Fourier transform infrared spectroscopy (FT‐IR) and Raman spectroscopy were employed to characterize the chemical bonds in Ir@NiFe‐MOF/NF. The FT‐IR spectra revealed shifts to higher frequencies in the carboxylate stretching vibration and M–O vibration peaks in Ir@NiFe‐MOF compared to those of NiFe‐MOF (Figure S13a,b, Supporting Information), indicating Ir bonding to the oxygen atoms on the surface of NiFe‐MOF.^[^ ^13^ ^]^ In addition, a broad band in the range of 500–600 cm^−1^ in the Raman spectra indicated the presence of Ni/Fe−O−Ir^[^ ^27^ ^]^ at the heterojunction interface of Ir@NiFe‐MOF (Figure S13c, Supporting Information). These results provide evidence for electron transfer from Ir NPs to NiFe‐MOF through the formation of Ni/Fe─O─Ir bonds at the heterojunction interface. The resulting modulation of the electronic structures of Ir and Ni/Fe are expected to accelerate HER and OER kinetics.

**Figure 3:**
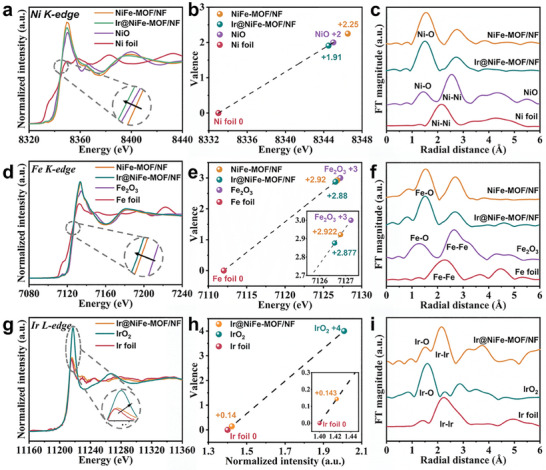
a) Normalized Ni K‐edge XANES spectra, b) fitted valence states and c) the corresponding FT‐EXAFS spectra in *R* space, d) normalized Fe K‐edge XANES spectra, e) fitted valence states and f) the corresponding FT‐EXAFS spectra in *R* space of Ir@NiFe‐MOF/NF, NiFe‐MOF/NF and the reference materials. g) Normalized Ir L_3_‐edge XANES spectra, h) fitted valence states, and i) the corresponding FT‐EXAFS spectra in *R* space of Ir@NiFe‐MOF/NF, Ir foil, and IrO_2_.

The X‐ray absorption fine structure (XAFS) analysis provided insights into the local electronic structure and coordination environment of Ni, Fe, and Ir in Ir@NiFe‐MOF/NF, allowing to further understand the electronic interaction between Ir NPs and NiFe‐MOF resulting from the formation of Ni/Fe─O─Ir bonding. X‐ray absorption near‐edge structure (XANES) and extended X‐ray absorption fine structure (EXAFS) tests of the Ni K‐edge, Fe K‐edge, and Ir L_3_‐edge were performed because XANES at the 3d element K‐edge and the 5d element L_3_‐edge is sensitive to the electronic structure.^[^ ^28^ ^]^ **Figure** **3**a illustrates the normalized Ni K‐edge XANES spectra of Ir@NiFe‐MOF/NF and NiFe‐MOF/NF with Ni foil and NiO as references. Ni K‐edge XANES spectra showed a negative shift and a significant reduction in Ir@NiFe‐MOF/NF compared to NiFe‐MOF/NF, indicating that the anchoring of Ir NPs on NiFe‐MOF increases the electron density around the Ni sites. However, the XANES spectrum of the Ni K‐edge of NiFe‐MOF has higher intensity and energy compared to that of NiO, which may be due to the higher valence state of Ni caused by Fe^3+^ doping in NiFe‐MOF. The average oxidation state (1.91) of Ni in Ir@NiFe‐MOF/NF, estimated from the threshold value (*E*_0_) obtained from the first derivative curves of the Ni K‐edge XANES^[^ ^29^ ^]^ (Figure 3b), was lower than that in NiFe‐MOF (2.25). These values again confirm the electron transfer of Ir NPs to NiFe‐MOF via Ni/Fe─O─Ir bonds, which is in agreement with the Ni 2p XPS analysis (negative shift). The EXAFS spectra of Ni in *R* space showed a negative shift in the primary peak attributed to the nearest shell coordination of the Ni─O bond of Ir@NiFe‐MOF (1.50 Å) compared to that in NiFe‐MOF (1.55 Å) (Figure 3c), indicating a strong interaction between immobilized Ir NPs and NiFe‐MOF/NF. The Fe K‐edge XANES spectra revealed a lower electron density of Fe in Ir@NiFe‐MOF compared to NiFe‐MOF (Figure 3d). The average valence state of Fe in Ir@NiFe‐MOF was ≈0.05 lower than that in NiFe‐MOF (Figure 3e). The EXAFS spectra in the Fe K‐edge indicated that the peaks in Ir@NiFe‐MOF and NiFe‐MOF were similar, with the dominant peak attributed to Fe─O bonds (Figure 3f). The Fe─O peak of Ir@NiFe‐MOF (1.55 Å) is negatively shifted by only 0.02 Å compared to that of NiFe‐MOF (1.57 Å), indicating that Ir NPs similarly alter the electronic structure of Fe through Fe─O─Ir bonds, which is also consistent with the Fe XPS results. The EXAFS fitting results of the Ni and Fe K‐edges showed that, compared to those of NiFe‐MOF/NF, the average bond lengths of Ni─O and Fe─O in Ir@NiFe‐MOF/NF were slightly reduced (Figure S14 and Table S3, Supporting Information), implying the formation of Ni/Fe─O─Ir bonds. The Ir L_3_‐edge XANES spectra showed that Ir in Ir@NiFe‐MOF/NF was mostly in a metallic state (Figure 3g), with an increase in intensity and a positive shift in the energy of the white line (WL) peak compared to the Ir foil, which is attributed to a decreased 5d occupation of Ir atoms in Ir@NiFe‐MOF/NF.^[^ ^30^ ^]^ The average valence state of Ir in Ir@NiFe‐MOF was ≈0.14, estimated by the intensity of the white line (WL) peak for Ir L_3_‐edge XANES, indicating charge transfer from Ir NPs to NiFe‐MOF (Figure 3h). The analysis was consistent with the Ir 4f XPS results showing a positive binding energy shift. Ir EXAFS analysis in *R* space revealed a dominant peak corresponding to Ir─Ir bonds, as well as Ir─O bonds, indicating the formation of a Ni/Fe─O─Ir bond between Ir NPs and NiFe‐MOF at the heterojunction interface (Figure 3i). Overall, taken together, the XANES, EXAFS, and XPS results confirmed the strong charge transfer between Ir NPs and NiFe‐MOFs through a unique interfacial Ni/Fe─O─Ir interaction.

### 2.2 Electrochemical Performance

**Figure 4:**
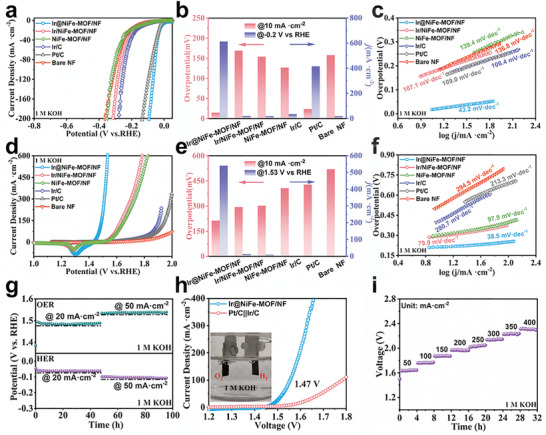
Electrochemical performance in 1.0 m KOH. a) Polarization curves, b) Comparison of the overpotentials at 10 mA cm^−2^ and the current densities at *η* = 200 mV, and c) the corresponding Tafel plots of all samples for HER. d) LSV curves e) Comparison of performance and f) corresponding Tafel slopes of the catalysts for OER; g) Time‐potential curves for HER and OER at 20 and 50 mA·cm^−2^. h) LSV curves of Ir@NiFe‐MOF/NF and Pt/C║Ir/C for overall water splitting. i) Time‐voltage curves for water electrolysis at different current densities.

The electrocatalytic performance of the synthesized electrocatalysts was assessed using a typical three‐electrode system with Hg/HgO as the reference electrode, a graphite rod as the counter electrode, and the as‐prepared self‐supporting electrodes as the working electrode. Electrochemical data for the electrocatalyst were collected in an N_2_‐saturated 1.0 m KOH solution at room temperature. Commercial Pt/C (20 wt.%) and Ir/C (5%) catalysts loaded on NF served as benchmarks. The electrocatalytic performance of Ir@NiFe‐MOF/NF electrodes with different Fe doping levels was first investigated. As shown in Figure S15 (Supporting Information), Ir@NiFex‐MOF/NF (*x* = 0, 0.1, 0.3, and 0.5) electrodes with different Fe contents exhibited similar HER performances. However, an overall enhancement was observed with increasing Fe doping level for their OER performance, indicating that the Fe doping was beneficial for the OER process. Despite this, Ir@NiFe_0.5_‐MOF/NF still exhibited a decreasing trend for OER performance, probably due to the disruption of the NiFe_0.5_‐MOF/NF ultra‐thin nanosheet array morphology. The electrochemical activity of Ir@NiFe‐MOF/NF with different Ir loadings was first investigated. Notably, Ir@NiFe‐MOF/NF prepared with 600 µL of IrCl_3_ exhibited the best catalytic activity for both the HER and OER (Figure S16, Supporting Information). We further examined the intrinsic activity of Ir@NiFe‐MOF/NF by calculating the mass activity, which revealed that Ir@NiFe‐MOF/NF prepared with 600 µL of IrCl_3_ had superior mass activity compared to higher Ir loadings. To assess the electrochemical surface area (ECSA) and the intrinsic activity of the electrocatalysts, we measured the double‐layer capacitance (*C*_dl_) by cyclic voltammetry (CV) curves (Figure S17, Supporting Information). Ir@NiFe‐MOF/NF‐600 displayed the largest electrochemical area, which decreased with further increasing in Ir content (Figure S18, Supporting Information), owing to Ir NPs agglomeration at higher concentrations. Excessive Ir loading resulted in a reduced number of accessible active sites and attenuated electrochemical activity.^[^ ^31^ ^]^ It is evident that like bare NF, pristine NiFe‐MOF/NF exhibited poor HER activity with an overpotential of 154 mV at 10 mA·cm^−2^ (**Figure** **4**a). In contrast, after the introduction of Ir NPs, Ir@NiFe‐MOF/NF required only an ultralow overpotential of 15 mV enabling a current density of 10 mA·cm^−2^, which surpasses that of commercial Pt/C (24 mV) and Ir/C (127 mV). These results not only emphasized the importance of electronic interactions between Ir and NiFe‐MOF in enhancing HER kinetics but also suggested that Ir NPs were the primary HER active sites, with NiFe‐MOF contributing negligibly to the HER electrocatalysis. It is worth noting that Ir/NiFe‐MOF/NF, where Ir NPs were physically loaded onto NiFe‐MOF/NF, did not exhibit enhanced HER activity (Figure 4b). This underscores the significance of the Ir NPs anchored on NiFe‐MOF/NF through Ir─O bonds. Furthermore, the overpotentials of Ir@NiFe‐MOF/NF at high current densities (100–800 mA·cm^−2^) were significantly lower than those of Pt/C (Figure S19, Supporting Information). Consequently, Ir@NiFe‐MOF/NF demonstrated a lower Tafel slope of 43.2 mV·dec^−1^ compared to Pt/C (109.0 mV·dec^−1^), Ir/C (106.4 mV·dec^−1^), and Ir/NiFe‐MOF/NF (107.1 mV·dec^−1^) (Figure 4c), indicating superior reaction kinetics for the HER.^[^ ^32^ ^]^

The OER performance of the electrocatalysts was also evaluated through linear sweep voltammetry (LSV) curves recorded from high to low potentials in 1.0 m KOH solution to avoid the effect of the capacitive current generated by the oxidation of Ni species. As illustrated in Figure 4d, Ir@NiFe‐MOF/NF exhibited excellent OER performance, achieving a low overpotential of 213 mV at 10 mA·cm^−2^, surpassing Ir/NiFe‐MOF/NF (294 mV) and NiFe‐MOF/NF (302 mV). It is noteworthy that despite the NiFe‐MOF's performance for OER being significantly lower than that of Ir@NiFe‐MOF, it still exhibited high OER activity. Therefore, besides Ir NPs, Ni, and Fe can also be considered as OER active sites. Notably, it delivered a current density of 540.4 mA·cm^−2^ at an overpotential of 300 mV, surpassing the performances of Ir/NiFe‐MOF/NF (12.8 mA·cm^−2^), NiFe‐MOF/NF (9.5 mA·cm^−2^), and Ir/C (1.7 mA·cm^−2^) by factors of 42.2, 56.9, and 317.9, respectively (Figure 4e). Particularly noteworthy, Ir@NiFe‐MOF/NF required only 291 and 317 mV overpotentials to achieve high current density operations of 400 and 800 mA·cm^−2^ (Figure S20, Supporting Information). Additionally, Ir@NiFe‐MOF/NF exhibited the fastest reaction kinetics (Figure 4f), as indicated by a Tafel slope of 38.5 mV·dec^−1^, outperforming Ir/NiFe‐MOF/NF (79.9 mV·dec^−1^) and NiFe‐MOF/NF (97.9 mV·dec^−1^). It is well known that the performance of catalysts is mainly determined by the intrinsic activity and the number of active sites. To further reveal the high intrinsic activity of Ir@NiFe‐MOF/NF, we assessed the electrochemical surface area (ECSA) by examining the double‐layer capacitance (*C*_dl_) (Figure S21, Supporting Information). Ir@NiFe‐MOF/NF exhibited the largest *C*_dl_ of 23.99 mF·cm^−1^, significantly exceeding NiFe‐MOF/NF (3.24 mF·cm^−1^) and Ir/NiFe‐MOF/NF (12.85 mF·cm^−1^), suggesting a higher number of active sites in Ir@NiFe‐MOF/NF for HER and OER^[^ ^33^ ^]^ The exceptional HER and OER properties of Ir@NiFe‐MOF/NF were further confirmed through electrochemical impedance spectroscopy (EIS) measurements. As depicted in Figure S22 (Supporting Information), Ir@NiFe‐MOF/NF demonstrated a much smaller charge transfer resistance than pristine NiFe‐MOF/NF and Ir/NiFe‐MOF/NF, indicating that the introduction of Ir NPs promoted electron transport, significantly enhancing the electrocatalytic performance for the HER and OER. Long‐term stability was evaluated through chronopotentiometric tests, which revealed that Ir@NiFe‐MOF/NF maintained its performance for over 48 h at current densities of 20 and 50 mA·cm^−2^ for the HER and OER, with no noticeable degradation in overpotential in an alkaline solution (Figure 4g). The polarization curves for HER and OER after stability testing clearly demonstrated negligible activity decay (Figure S23, Supporting Information), indicating the outstanding electrochemical stability of Ir@NiFe‐MOF/NF.

The exceptional catalytic activities demonstrated by Ir@NiFe‐MOF/NF for both the HER and the OER inspired us to assemble an electrolyzer with Ir@NiFe‐MOF/NF serving as both the cathode and anode to further assess its practical potential for overall water splitting. As depicted in Figure 4h, Ir@NiFe‐MOF/NF exhibited an impressively low cell voltage of 1.47 V, driving a current density of 10 mA·cm^−2^. This voltage is significantly lower than that of Pt/C║Ir/C (1.57 V) and even outperforms recently reported bifunctional electrocatalysts (Tables S4–S6, Supporting Information). These results underscore the outstanding activity of Ir@NiFe‐MOF/NF in facilitating overall water splitting. To evaluate its long‐term durability, we further conducted continuous operation at a constant voltage of 1.6 V (Figure S24, Supporting Information). The results showed that the current density fluctuated within a limited range for 90 h without significant performance degradation. Most notably, Ir@NiFe‐MOF/NF maintained its high activity for 32 h in a large current density range of 50–400 mA·cm^−2^ (Figure 4i), indicating its promising potential for practical applications.

To gain insights into the structural stability of Ir@NiFe‐MOF/NF under alkaline electrolytic conditions, a series of characterization techniques, including SEM, TEM, PXRD, Raman, and XPS, were employed to examine the fate of the catalyst after HER and OER testing (Figures S25–S27, Supporting Information). The SEM and TEM analyses revealed that the surface morphology of Ir@NiFe‐MOF/NF remained remarkably stable after stability testing. The preservation of the catalyst's surface integrity underscored its robust structural stability under operational conditions. However, the PXRD analysis disclosed the presence of surface‐reconstructed NiFeOOH during the OER process, and a minor fraction of iron in the original NiFe‐MOF may be incorporated into NiOOH to form Fe‐doped NiOOH (NiFeOOH).^[^ ^34^ ^]^ This observation was corroborated by its Raman spectrum, which confirmed the formation of metal oxyhydroxide (NiFeOOH).^[^ ^35^ ^]^ The EDS spectra and XPS survey spectra further affirmed the presence of Ir, Ni, Fe, C, and O in the catalyst before and after electrolysis (Figures S28 and S29, Supporting Information), indicating the persistence of the catalyst's elemental composition. Notably, the appearance of peaks attributed to Ni^3+^ in the Ni 2p spectrum and a positive shift in Fe^3+^ binding energy in the Fe 2p spectrum provided conclusive evidence for the generation of NiFeOOH during OER (Figure S29c,d, Supporting Information). Additionally, Raman analysis revealed a partial conversion of Ir@NiFe‐MOF/NF to Ni(OH)_2_ during the HER in an alkaline solution.^[^ ^36^ ^]^ Despite these alterations in the material composition, XPS results indicated that there was no significant valence change in these elements after HER. Therefore, these comprehensive findings underscore that the meticulous design and fabrication of Ir@NiFe‐MOF/NF electrodes result in an effective bifunctional electrocatalyst for efficient hydrogen (H_2_) and oxygen (O_2_) production through water splitting. The observed structural changes during OER and HER processes highlight the dynamic nature of the catalyst's surface under electrochemical conditions, contributing to a more nuanced understanding of its electrocatalytic performance.

### 2.3 Theoretical Calculations

**Figure 5:**
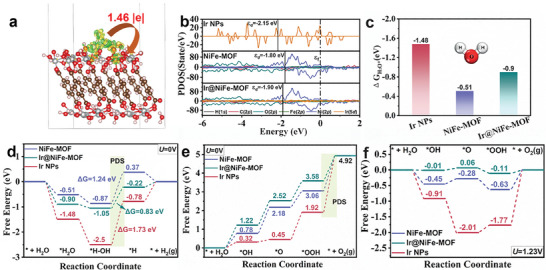
a) The charge density difference of Ir@NiFe‐MOF (the yellow and green colors represent charge accumulation and depletion, respectively). b) The PDOS of Ir NPs, NiFe‐MOF, and Ir@NiFe‐MOF (the dotted line represents the Fermi level (*ε*_f_), and solid line is the position of d‐band center (*ε*_d_)). c) ^*^H_2_O adsorption free energy. d) Calculated free energy diagram for HER at *U* = 0.0 V. Calculated free energy diagram at *U* = 0.0 V e) and *U* = 1.23 V f) for OER.

To gain deeper insights into the influence of the interaction between Ir NPs and NiFe‐MOF on the electrochemical activities of the HER and the OER, density functional theory (DFT) calculations were conducted. The optimized models of Ir NPs (Ir_5_ cluster), NiFe‐MOF, and Ir@NiFe‐MOF are shown in Figure S30 (Supporting Information). The charge density difference (CDD) shows that the Ir_5_ cluster undergoes both electron accumulation (as indicated by the yellow region) and dissipation (green region) in **Figure** **5**a. This dual electron transfer indicates a bidirectional electronic interaction between Ir_5_ cluster and NiFe‐MOF. The Ir_5_ cluster transfers 1.46 |e| electrons to NiFe‐MOF based on the Bader charge calculation, consistent with the findings from XPS and XANES analyses. These results provide further evidence that the electronic structure of the heterojunction interface is finely tuned after anchoring the Ir NPs onto NiFe‐MOF via Ir─O─Ni/Fe bonds.^[^ ^37^ ^]^ In addition, as shown in Figure S31 (Supporting Information), electronic states of Ir@NiFe‐MOF and NiFe‐MOF were continuously distributed around the Fermi level (*ε*_f_), and both exhibited zero bandgaps, indicating their metallic characteristics. Notably, the total density of states (DOS) at near the Fermi level in Ir@NiFe‐MOF exceeded that of Ir NPs and NiFe‐MOF, indicating that the heterostructure constructed with Ir NPs and NiFe‐MOF through Ir─O─Ni/Fe bonding exhibited enhanced conductivity compared to either monomer,^[^ ^38^ ^]^ consistent with the EIS results. Furthermore, a lower d‐band center (*ε*_d_) indicates a stronger adsorption ability for the intermediates.^[^ ^39^ ^]^ As depicted in Figure 5b, the *ε*_d_ values of Ir@NiFe‐MOF were −1.90 eV, higher than that of Ir NPs (−2.15 eV) but lower than that of NiFe‐MOF (−1.80 eV). This indicates that anchoring Ir NPs on NiFe‐MOF modified the electronic structure and led to a suitable adsorption‐free energy for intermediates.^[^ ^40^ ^]^ This adjustment perhaps accelerated the adsorption and desorption processes on the catalyst surface, resulting in the superior electrocatalytic activity of Ir@NiFe‐MOF.

Water adsorption is a key step for HER in basic HER process, and strong water adsorption is beneficial to the formation of ^*^H intermediate in the subsequent step. The adsorption‐free energy of water molecules is −1.48, −0.90, and −0.51 eV on the Ir_5_ cluster, Ir@NiFe‐MOF, and NiFe‐MOF (Figure 5c), respectively. This result shows that the water molecule adsorption was enhanced upon introducing Ir NPs in NiFe‐MOF.^[^ ^41^ ^]^ The free energy diagrams of the HER are presented in Figure 5d and optimized intermediate structures are illustrated in Figures S32–S34 (Supporting Information). The potential‐determining step (PDS) for HER on the Ir_5_ cluster, Ir@NiFe‐MOF, and NiFe‐MOF is the formation of the ^*^H intermediate with a change of free energy (ΔG) of 1.73, 0.83, and 1.24 eV. The ΔG_*H_ of Ir@NiFe‐MOF (−0.22 eV) is closest to 0 compared with those of Ir NPs (−0.78 eV) and NiFe‐MOF (0.37 eV). These results demonstrate that the introduction of Ir NPs in NiFe‐MOF affects H_2_O molecule and ^*^H adsorption,^[^ ^42^ ^]^ resulting in excellent HER activity. Furthermore, the free energy diagrams of OER are displayed in Figure 5e (*U* = 0.0 V) and Figure 5f (*U* = 1.23 V), respectively. The PDS for the three catalysts is the final process of O_2_ release. It was found that Ir@NiFe‐MOF exhibits the lower free energy barrier of 1.34 eV compared with the Ir_5_ clusters (3.0 eV) and NiFe‐MOF (1.86 eV) in Figure 5e. At an equilibrium potential of OER (*U* = 1.23 V), Ir@NiFe‐MOF also has the lowest overpotential of 0.11 V among the three catalysts. These findings highlight the constructive role of the Ir─O─Ni/Fe bond at the heterojunction interface between Ir NPs and NiFe‐MOF. This interface facilitates charge transfer, adjusts the electronic structure, optimizes the adsorption of intermediates, and reduces the energy barriers, thereby enhancing the electrocatalytic activity of the HER and OER processes.

In accordance with PXRD and Raman results, the surface of Ir@NiFe‐MOF/NF could be reconstructed to NiFeOOH species during the OER process. Subsequently, DFT calculations were performed for Ir@NiFeOOH and NiFeOOH (Figures S35,S36, Supporting Information) to investigate the electrocatalytic enhancement mechanism of the reconstructed structures. Figure S37 (Supporting Information) illustrates the free energy diagram calculated at *U* = 0 V and *U* = 1.23 V, confirming O_2_ release as the PDS for both Ir NPs and NiFeOOH, with the PDS for Ir@NiFeOOH being the formation of ^*^OOH. It is suggested that the transformation structure (Ir@NiFeOOH) of Ir@NiFe‐MOF is also conducive to the release of O_2_. The ΔG value of the PDS for Ir@NiFeOOH (2.01 eV) is lower than that of NiFeOOH (2.14 eV) and Ir NPs (3.0 eV) (Figure S37c, Supporting Information). And the lowest theoretical overpotential of the PDS (the formation of ^*^OOH) for Ir@NiFeOOH is 0.78 V at *U* = 1.23 V (Figure S37d, Supporting Information), surpassing NiFeOOH (1.18 V) and Ir NPs (1.77 V). However, the PDS of Ir@NiFe‐MOF is only 0.83 eV with a theoretical overpotential of 0.11 V (Figure 5), markedly lower than Ir@NiFeOOH (2.01 eV and 0.78 V), suggesting that the OER activity of Ir@NiFe‐MOF is superior to Ir@NiFeOOH. These DFT results reveal that the remarkable electrocatalytic performance of the catalyst mainly stems from electronic interactions between Ir NPs and NiFe‐MOF, and the reconstructed Ir@NiFeOOH also makes due contributions to the overall OER activity if any.

## 3 Conclusion

This work has described new HER and OER bifunctional catalysts with significantly improved electrochemical performance, obtained by anchoring Ir NPs on the surface of NF self‐supporting ultrathin NiFe‐MOF nanosheets and resulting from the galvanic reduction of Ir^3+^ by NF. The Ir@NiFe‐MOF/NF catalyst displayed remarkable electrochemical performances, outperforming commercial Pt/C and Ir/C benchmarks. It achieved a low overpotential of only 15 mV at 10 mA·cm^−2^ for the HER and required overpotentials of 213 mV for the OER to reach a current density of 10 mA·cm^−2^ in 1 m KOH, respectively. Using Ir@NiFe‐MOF/NF as both the cathode and anode, only battery voltage of 1.47 V was needed to drive a current density of 10 mA·cm^−2^ under alkaline conditions, showing excellent overall water splitting performance. Furthermore, the FT‐IR and Raman spectra confirmed that Ir NPs are dispersed on the surface of the NiFe‐MOF nanosheets and form Ir─O─Ni/Fe bonding with the surface oxygen atoms of the MOF. XPS results showed strong electronic interactions between Ni/Fe and Ir NPs and partial charge transfer from Ir NPs to NiFe‐MOF, which was also confirmed by EXAFS and XANES. DFT calculations demonstrated that the introduction of Ir NPs can facilitate the adsorption of H_2_O, shifting the d‐band center position by adjusting the electronic structure, optimizing the adsorption of HER and OER intermediates, and promoting the final release of H_2_ and O_2_. These factors collectively contributed to the enhanced electrocatalytic water‐splitting performance. The in‐depth studies performed in this work of the interactions between Ir nanoparticles and MOF should provide a valuable contribution to the design of efficient electrocatalysts for overall water splitting.

## 4 Experimental Section

### Preparation of NiFe‐MOF/NF

Ni(NO_3_)_2_·6H_2_O (29.1 mg, 0.1 mmol) and FeCl_3_·6H_2_O (8.1 mg, 0.03 mmol) were dissolved in 3.0 mL deionized water. This solution was then mixed with a 9.0 mL N,N‐dimethylformamide (DMF) solution containing 16.6 mg terephthalic acid (1,4‐BDC) (0.1 mmol). After it was stirred for 30 min, the solution was transferred into a 20 mL Teflon‐lined autoclave. The dried NF was placed in the autoclave, as shown in Scheme 1, which was sealed and heated at 150 °C for 6 h. After cooling, the sample was removed and thoroughly washed three times alternately with water and ethanol, to eliminate impurities and the NiFe‐MOF powder that did not grow on the NF substrate. The final step involved drying the sample at 60 °C overnight.

### Preparation of Ir@NiFe‐MOF/NF

IrCl_3_·xH_2_O (29.8 mg, 0.1 mmol) was completely dissolved at 80 °C in 2 mL deionized water, forming a 0.05 m IrCl_3_ solution. NiFe‐MOF/NF was placed into an autoclave containing 600 µL of the 0.05 m IrCl_3_ solution, adjusting the total volume to 10 mL with deionized water. The autoclave was sealed and heated at 80 °C for 12 h. After cooling, the sample was removed and subjected to three successive washings with water and ethanol. Subsequently, the sample was dried at 50 °C in a vacuum oven. Varying the amount of 0.05 m IrCl_3_ solution (200, 400, 800, and 1000 µL) allowed for the preparation of Ir@NiFe‐MOF/NF samples with different Ir loadings.

Electrochemical measurements for all the samples were conducted using a CHI660E electrochemical workstation with a standard three‐electrode system. Density functional theory (DFT) calculations were carried out using the Vienna Ab Initio software package (VASP). Details of the electrochemical tests, structural characterizations, and theoretical calculations are provided in the Supporting Information.

## Conflict of Interest

The authors declare no conflict of interest.

## Supporting information

Supporting Information

## Data Availability

The data that support the findings of this study are available in the supplementary material of this article.
