# Supplementary material for: Interfacial Electronic Interactions Between Ultrathin NiFe‐MOF Nanosheets and Ir Nanoparticles Heterojunctions Leading to Efficient Overall Water Splitting
Source: Adv Sci (Weinh). 2024 Apr 26;11(28):2401780. doi: 10.1002/advs.202401780 (PMC11267393; doi:10.1002/advs.202401780)
Supplement: Supplementary file 1 — Supporting Information [file ADVS-11-2401780-s001.pdf]

ADVANCED SCIENCE
Open Access

Supporting Information

for *Adv. Sci.*, DOI 10.1002/advs.202401780

Interfacial Electronic Interactions Between Ultrathin NiFe-MOF Nanosheets and Ir Nanoparticles Heterojunctions Leading to Efficient Overall Water Splitting

Cong Li, Wei Zhang, Yongyong Cao, Jun-Yang Ji, Zhao-Chen Li, Xu Han, Hongwei Gu, Pierre Braunstein and Jian-Ping Lang**

Supporting Information

Interfacial Electronic Interactions between Ultrathin NiFe-MOF Nanosheets and Ir Nanoparticles Heterojunctions Leading to Efficient Overall Water Splitting

Cong Li, Wei Zhang, Yongyong Cao,* Jun-Yang Ji, Zhao-Chen Li, Xu Han, Hongwei Gu, Pierre Braunstein, Jian-Ping Lang*

C. Li, W. Zhang, J. Y. Ji, Z. C. Li, X. Han, H. Gu, J.-P. Lang
College of Chemistry, Chemical Engineering and Materials Science, Soochow University, Suzhou, 215123, Jiangsu, People's Republic of China
E-mail: jplang@suda.edu.cn

C. Li, J.-P. Lang
State Key Laboratory of Organometallic Chemistry, Shanghai Institute of Organic Chemistry, Chinese Academy of Sciences, Shanghai 200032, People's Republic of China

Y. Cao
College of Biological, Chemical Science and Engineering, Jiaxing University, Jiaxing, 314001, Zhejiang, People's Republic of China
E-mail: cyy@zjxu.edu.cn

P. Braunstein
Université de Strasbourg - CNRS, Institut de Chimie (UMR 7177 CNRS), 4 rue Blaise Pascal-CS 90032, 67081 Strasbourg, France

Table of Contents

1. Experimental Section S4
Materials S4
Preparation of Ir@NiFe-MOF/NF electrode with different amounts of Fe doping S4
Preparation of Ir/NiFe-MOF/NF electrode S4
Preparation of Pt/C electrode S4
Preparation of Ir/C electrode S4
Characterization S4
Electrochemical measurements S5
Theoretical calculations S5
2. Supplementary Figures S7
Figure S1. (a) PXRD patterns, (b, c) SEM images and (d) EDS mapping of NiFe-MOF/NF. S7
Figure S2. (a) HAADF image and EDS mapping and (b) EDS spectra of NiFe-MOF. S7
Figure S3. The photographs of (a) bare NF, (b) NF-H_2O, (c) NF-Ir, (d) the solution after the reaction of NF and $IrCl_3$, and (e) the solution after the reaction of NF and H_2O; the SEM images of (f) bare NF, (g) NF-H_2O and (h) NF-Ir; the EDX spectra of (i) bare NF, (j) NF-H_2O and (k) NF-Ir. S9
Figure S4. Diagram of the atomic arrangement of NiFe-MOF. S10
Figure S5. The cross-sectional SEM images of Ir@NiFe-MOF/NF. S10
Figure S6. The EDS mapping and the corresponding EDS spectra of Ir@NiFe-MOF/NF. S11
Figure S7. PXRD patterns of (a) Ni-MOF and NiFe-MOF with different amounts of Fe doping, (b) PXRD patterns of Fe-MOF. S11
Figure S8. SEM images and EDS spectra of Ni-MOF/NF, $NiFe_{0.1}$-MOF/NF, $NiFe_{0.5}$-MOF/NF and Fe-MOF/NF. S12
Figure S9. SEM images and EDS spectra of Ir@Ni-MOF/NF, Ir@$NiFe_{0.1}$-MOF/NF, and Ir@$NiFe_{0.5}$-MOF/NF. S13
Figure S10. PXRD patterns of (a) NiFe-MOF/NF and Ir@NiFe-MOF/NF and (b) Ir@NiFe-MOF/NF-200/400/600/800/1000. .... S13
Figure S11. SEM images and EDS spectra of Ir@NiFe-MOF/NF-200/400/600/800/1000. .... S14
Figure S12. XPS survey spectra (a, b) and High-resolution XPS spectra of the Fe 2p (c) and C 1s (d) of NiFe-MOF/NF and Ir@NiFe-MOF/NF. S15
Figure S13. FT-IR spectra (a, b) and Raman spectra (c)of NiFe-MOF/NF and Ir@NiFe-MOF/NF. S15
Figure S14. EXAFS fitting spectra of the Ni and Fe K-edge for NiFe-MOF/NF and Ir@NiFe-MOF/NF. S16
Figure S15. LSV Polarization curves for HER (a) and OER (b) of Ir@Ni-MOF/NF, Ir@$NiFe_{0.1}$-MOF/NF, Ir@$NiFe_{0.3}$-MOF/NF and Ir@$NiFe_{0.5}$-MOF/NF. S16
Figure S16. (a) HER polarization curves, (b) the mass-normalized LSV curves, (c) OER polarization curves and (d) the mass-normalized LSV curves of Ir@NiFe-MOF/NF-200/400/600/800/1000 in 1.0 M KOH. S17
Figure S17. Electrochemical cyclic voltammetry curves for Ir@NiFe-MOF/NF with different Ir content at different scan rates in 1.0 M KOH. S17
Figure S18. The capacitive currents as a function of the scan rates (a). The double-layer capacitance (C_{dl}) values of different samples (b). S18
Figure S19. (a) the HER polarization curves of samples in the large-current ranges and (b) the overpotential comparison of Ir@NiFe-MOF/NF and Ir/NiFe-MOF/NF at various current densities. S18
Figure S20. The OER polarization curves of samples in the large-current ranges (a) and the overpotential comparison of Ir@NiFe-MOF/NF and Ir/NiFe-MOF/NF at various current densities (b). S18
Figure S21. CV curves of (a) Ir@NiFe-MOF/NF, (b) Ir/NiFe-MOF/NF, and (c) NiFe-MOF/NF at increasing scan rates from 10-100 $mA\cdot s^{-1}$ in 1.0 M KOH and (d)The double-layer capacitance (C_{dl}) values of different samples. S19
Figure S22. Nyquist plots measured at the potential of (a, b) -30 mV for HER and (c) 1.53 V for OER in 1.0 M KOH. S19
Figure S23. The LSV curves of Ir@NiFe-MOF/NF before and after (a) HER and (b) OER tests. S20
Figure S24. The i-t curves of Ir@NiFe-MOF/NF with 1.6 V toward overall water splitting. S20
Figure S25. SEM (a, b) and TEM (c, d) images of Ir@NiFe-MOF/NF after HER in 1 M KOH. S20
Figure S26. SEM (a, b) and TEM (c, d) images of Ir@NiFe-MOF/NF after OER in 1 M KOH. S21
Figure S27. PXRD patterns (a) and Raman spectra (b) of Ir@NiFe-MOF/NF after HER and OER in 1 M KOH. S21
Figure S28. The EDS spectra of Ir@NiFe-MOF/NF after (a) HER and (b) OER in 1 M KOH. S22
Figure S29. (a) XPS survey spectra and High-resolution XPS spectra of Ir 4f (b), Ni 2p (c), and Fe 2p (d) of Ir@NiFe-MOF/NF after HER and OER in 1 M KOH. S22
Figure S30. Structural models of (a) NiFe-MOF and (b) Ir@NiFe-MOF and (c) Ir NPs. S23
Figure S31. The DOS plots for Ir NPs, NiFe-MOF and Ir@NiFe-MOF. The dashed line indicates the Fermi level for each system. S23
Figure S32. The optimized adsorption structures of HER and OER process intermediates at Ir NPs. S23
Figure S33. The optimized adsorption structures of HER and OER process intermediates at NiFe-MOF S24
Figure S34. The optimized adsorption structures of HER and OER process intermediates at Ir@NiFe-MOF. S24
Figure S35. The optimized adsorption structures of OER process intermediates at NiFeOOH S25

Figure S36. The optimized adsorption structures of OER process intermediates at Ir@NiFeOOH. S25
Figure S37. (a, b) Reaction pathways in the OER process, and (c, d) corresponding free energy difference for Ir NPs, NiFeOOH and Ir@NiFeOOH. S26
3. Supplementary Tables S26
Table S1. The compositions of the pristine NiFe-MOF/NF and Ir@NiFe-MOF/NF with different amounts of $IrCl_3$ determined by ICP-OES. S26
Table S2. The weight of Ir@NiFe-MOF can be estimated by measuring the difference in mass after stripping it off from the NF through high-power sonication. The area of Ir@NiFe-MOF/NF is 2 cm × 2 cm. S27
Table S3. EXAFS fitting parameters at the Ni and Fe K-edge for NiFe-MOF/NF and Ir@NiFe-MOF/NF. S27
Table S4. Comparison of Ir@NiFe-MOF/NF with the reported electrocatalysts for HER performance in alkaline. S28
Table S5. Comparison of Ir@NiFe-MOF/NF with the reported electrocatalysts for OER performance in alkaline. S29
Table S6. Comparison of Ir@NiFe-MOF/NF with the reported electrocatalysts for overall water splitting in alkaline. S30
References S30

1. Experimental Section

Materials

Nickel nitrate hexahydrate ($Ni(NO_3)_2 \cdot 6H_2O$), iron chloride hexahydrate ($FeCl_3 \cdot 6H_2O$), iridium trichloride hydrate ($IrCl_3 \cdot xH_2O$), terephthalic acid (1,4-H_2BDC), sodium dihydrogen phosphate (NaH_2PO_4), disodium hydrogen phosphate (Na_2HPO_4), potassium hydroxide (KOH), isopropanol and N,N-dimethylformamide (DMF) were purchased from Sinopharm Group Chemical Reagent Co., Ltd.. 5% Nafion, 20 wt% platinum carbon (Pt/C) and 5 wt% iridium carbon (Ir/C) were purchased from Sigma-Aldrich. All chemicals were directly used without further purification. The Ni foam (NF) substrate (2.0 cm × 2.0 cm × 0.5 mm) was first carefully sonicated in 3 M HCl for 10 min, followed by rinsing with water, ethanol, and acetone to clean its surface.

Preparation of Ir@NiFe-MOF/NF electrode with different amounts of Fe doping

The preparation of Ir@NiFex-MOF/NF with different amounts of Fe doping (x = 0, 0.1, 0.5) was similar to that of Ir@NiFe-MOF/NF. During the synthesis of MOF/NF precursors, the amount of Fe was changed to 0 mmol, 0.01 mmol, 0.05 mmol, and 0.13 mmol, and the total molar amount of Ni and Fe was kept at 0.13 mmol. The as-prepared samples were labeled as Ir@Ni-MOF/NF, Ir@$NiFe_{0.1}$-MOF/NF, Ir@$NiFe_{0.5}$-MOF/NF and Ir@Fe-MOF/NF, respectively.

Preparation of Ir/NiFe-MOF/NF electrode

The Ir/C (5.0 mg) was dispersed into 500 µl of solution containing a mixture of 485 µl of isopropanol, 15 µl of 5% Nafion. The 50 µl catalyst ink was then drop-dried onto the NiFe-MOF/NF and dried for electrocatalytic analysis.

Preparation of Pt/C electrode

The Pt/C (2.5 mg) was dispersed into 500 µl of solution containing a mixture of 485 µl of isopropanol, 15 µl of 5% Nafion. The 25 µl catalyst ink was then drop-dried onto the NF and dried for electrocatalytic analysis.

Preparation of Ir/C electrode

The Ir/C (5.0 mg) was dispersed into 500 µl of solution containing a mixture of 485 µl of isopropanol, 15 µl of 5% Nafion. The 50 µl catalyst ink was then drop-dried onto the NF and dried for electrocatalytic analysis.

Characterization

Powder X-ray diffraction (PXRD) measurements were performed on an X'Pert-Pro MPD diffractometer (PANalytical, Netherlands) using a Cu Kα X-ray source (λ = 1.540598 Å). Scanning electron microscopy (SEM) and energy-dispersive X-ray spectroscopy (EDS) images were obtained by cold field emission scanning electron microscopy (Hitachi S-4700). High-resolution transmission electron microscopy (HRTEM), high-angle annular dark-field scanning transmission electron

microscopy (HAADF-STEM), and elemental mapping were examined by an FEI Talos transmission electron microscope (Thermo). Raman spectra were recorded on a LabRAM Soleil high-resolution Fast Raman Spectroscopy excited by a 638 nm laser. Fourier Transform Infrared Spectra (FT-IR) were obtained by an Infrared spectrometer (Nicoletis, Thermo). The Ir, Ni and Fe contents in Ir@NiFe-MOF/NF were quantitatively measured by ICP–OES using a Bruker 5110 instrument. Atomic force microscopy (AFM) imaging was performed on a Bruker Dimension Icon. X-ray photoelectron spectra (XPS) were acquired with a Thermo EXCALAB 250 XI instrument. X-ray absorption fine structures (XAFS) of the Ni and Fe K-edge and Ir L_{III}-edge were measured using an easyXAFS300+ instrument.

Electrochemical measurements

All electrochemical tests were conducted on a CHI 660E electrochemical workstation using a typical three-electrode system. The working electrode was the as-fabricated NF self-supporting electrocatalyst electrode (1.0 cm × 0.5 cm), the counter electrode was a graphite carbon rod, and the reference electrode was an Hg/HgO electrode. A 1.0 M KOH and PBS aqueous solution were used as the electrolyte. Prior to the electrochemical measurement, the electrolyte was purged with high-purity N_2 for 30 min. Linear sweep voltammetry (LSV) curves were obtained at a scan rate of 2 $mV \cdot s^{-1}$ with 95% iR compensation. Electrochemical impedance spectroscopy (EIS) measurements were performed in the frequency range of 100 kHz–0.1 Hz with an amplitude of 5 mV. Electrochemical surface area (ECSA) was calculated from cyclic voltammetry (CV) measurements in the non-Faradaic potential region at different scan rates (10, 20, 30, 40, 60, 80, and 100 $mV \cdot s^{-1}$) to determine the electrochemical double-layer capacitance (C_{dl}). The reference electrode was calibrated to the reversible hydrogen electrode (RHE): $E_{RHE} = E_{Hg/HgO} + 0.098\ V + 0.059 \times pH$).

Theoretical calculations

All calculations were performed by a spin-polarized density functional theory (DFT) approach using the DMol3 code [1,2]. The projector augmented-wave (PAW) potentials were adopted to describe the electron correlation interactions, [3,4] and the Perdew, Burke, and Ernzerhof (PBE) within the generalized gradient approximation (GGA) approach was employed [5,6]. The DFT-D3 was considered to correct the interaction of van der Waals forces in all computation [7]. A vacuum space exceeds 15.00 Å was employed to avoid the interaction between two periodic units. On the plane-wave basis of electron convergence with an energy cutoff of 520 eV, the convergence criteria for force and energy were set to 0.05 eV/Åand 10^{-5} eV of the total energy with respect to the calculation accuracy, respectively. The Brillouin zones were sampled with a K-point grid of 3 × 3 × 1 for all geometry optimization and electronic properties calculation. The electron charge transfer between substrate and adsorbates was calculated using the Bader charge analysis method [8,9]. The correction term of the

Gibbs free energy change (ΔG) under standard conditions (298.15 K and 0.1 MPa) was employed in the computational hydrogen electrode (CHE) model proposed by Nørskov et al. [10].

In this regard, the Gibbs free energy change of each step was obtained by the equation: $\Delta G = \Delta E + \Delta E_{ZPE} + \int CpdT - T\Delta S + \Delta G_U$, where ΔE the free energy change that can be obtained from DFT calculations, ΔE_{ZPE} and ΔS are the zero-point correction energy and entropy gradient, respectively; $\int CpdT$ is the enthalpic temperature correction, and considering at a temperature of 298.15 K. $\Delta G_U = eU$, is the electrode potential contribution.

The applied potential (U) was defined as the maximum Gibbs free energy change ($-\Delta G_{max}/e$) along the reaction path, and to ensure that each elementary reaction step was exergonic, it was determined by the potential-determining step (PDS).

2. Supplementary Figures

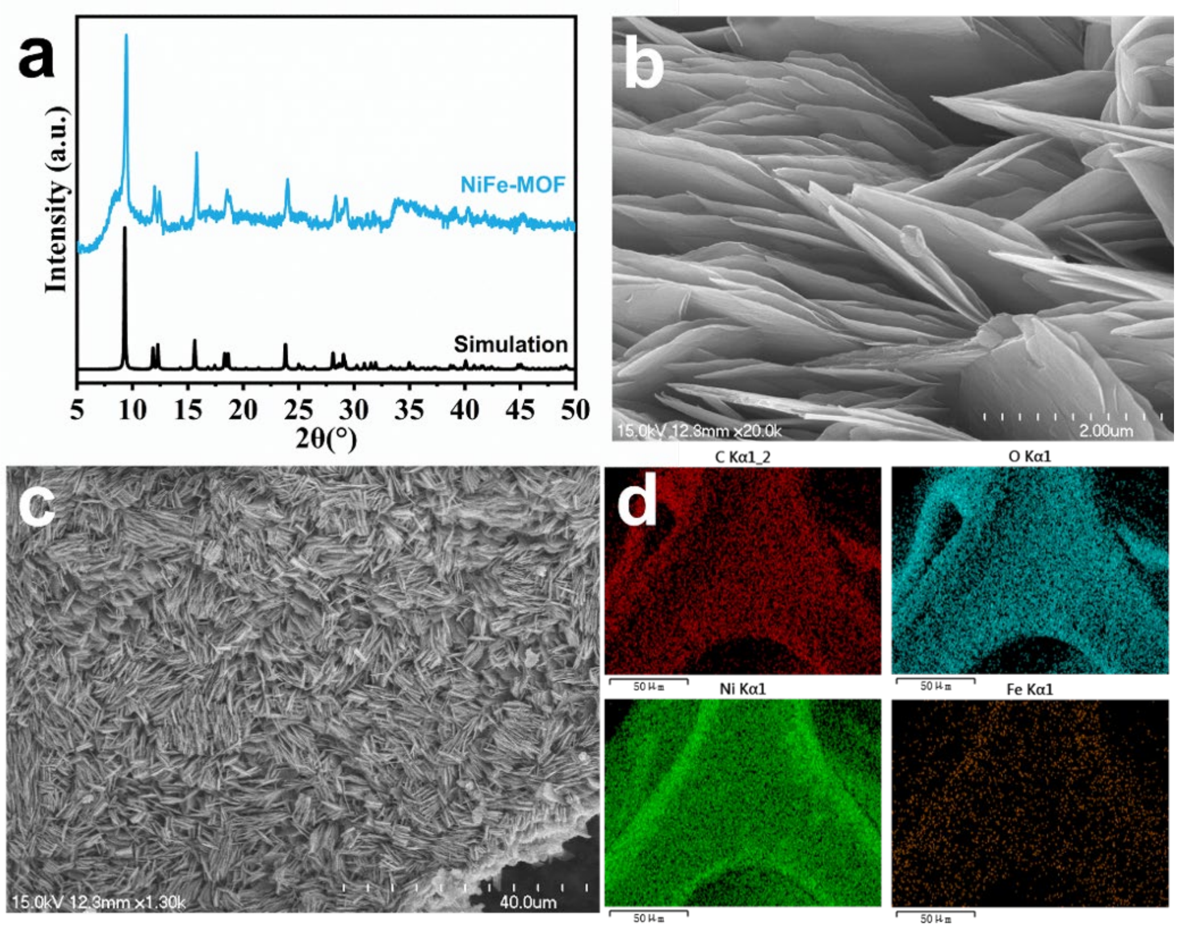

Figure S1. (a) PXRD patterns, (b, c) SEM images and (d) EDS mapping of NiFe-MOF/NF.

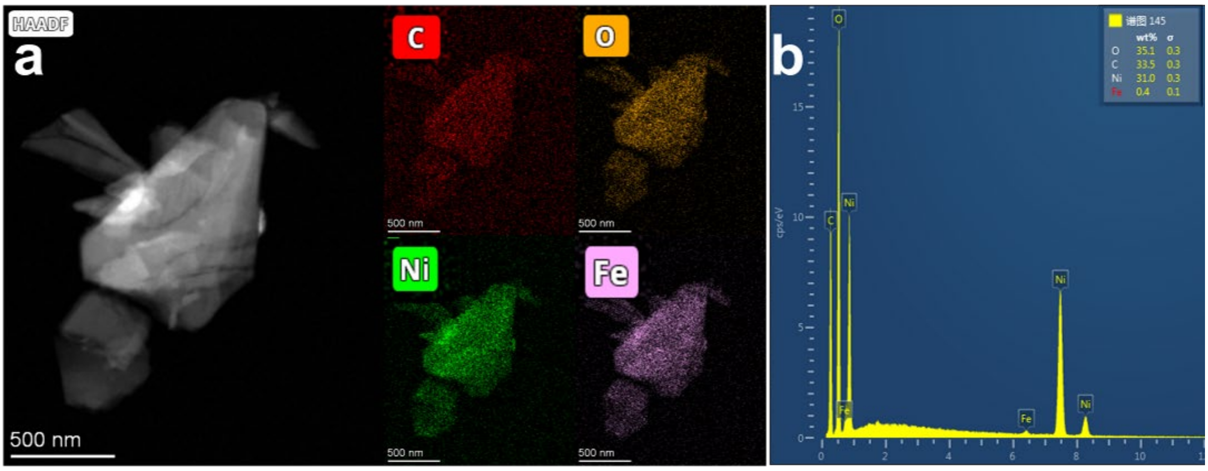

Figure S2. (a) HAADF image and EDS mapping and (b) EDS spectra of NiFe-MOF.

Mechanism of Ir nanoparticles formation by reduction of Ir^{3+} by nickel foam:

To elucidate the mechanism of the reduction of Ir^{3+} and the formation of Ir NPs, we have designed a series of comparative experiments as follows:

1. Pristine nickel foam (NF) before reaction (bare NF).
2. Nickel foam was immersed into H_2O at 80°C for 12 hours (NF-H_2O).
3. Nickel foam was immersed into H_2O solution containing 15 mg $IrCl_3$ at 80°C for 12 hours (NF-Ir).
4. The H_2O solution containing 15 mg $IrCl_3$ was maintained at 80°C for 12 hours.

SEM and EDS were employed to characterize the morphologies and surface chemical compositions of the immersed NF samples. The solution states of control groups 3 and 4 were analyzed with ICP-OES, which determined the concentration of Ni^{2+} dissolved in the reaction solution during the reaction of NF in the solution of control groups 2 and 3.

The results revealed that the image of the NF-H_2O sample resembled that of bare NF, exhibiting metallic color, while the surface of the NF-Ir sample was covered with a layer of black substance (**Figure S3a-c**). Observation of the reaction solution of the NF-Ir sample and the solution after reacting with pure $IrCl_3$ revealed that the reaction solution of NF-Ir was transparent with slight suspended particles, exhibiting a distinct Tyndall effect, whereas the treated $IrCl_3$ solution remains a yellow-orange solution (color of Ir^{3+} aqueous solution) without significant Tyndall effect (**Figure S3d-e**). This confirmed that the reaction between NF and Ir^{3+} yielded Ir NPs, and the $IrCl_3$ solution remained unchanged even after prolonged heating.

SEM images showed that the surface of the NF-H_2O sample exhibits nanosheets resembling hydroxides formed through the hydrolysis of Ni^{2+} during the hydrothermal process, whereas the NF-Ir sample exhibited apparent coverage of Ir NPs on the NF surface (**Figure S3f-h**). EDX analysis revealed that the elemental composition of bare NF surface is 99.4 wt% Ni and 0.6 wt% O, while Ni-H_2O consisted of 96.9 wt% Ni and 3.1 wt% O. Notably, the elemental ratio in NF-Ir was 80.1 wt% Ni and 19.9 wt% Ir, with negligible O content (**Figure S3i-k**). These results clearly demonstrated that Ir^{3+} was reduced by the metallic Ni(0) of the NF substrate, forming Ir NPs. According to the ICP-OES results, the content of Ni in the mother solution of the NF-Ir sample was much higher than that of the NF-H_2O sample, indicating the release of the oxidized species (Ni^{2+}) into the solution after the formation of the NF-Ir sample.

These findings supported our proposed mechanism: the metallic Ni(0) in the NF substrate reduces Ir^{3+} ions to form Ir NPs, with the resulting oxidized species (Ni^{2+}) being released into the reaction solution. The generated Ir(0) atoms are then anchored on the surface of NiFe-MOF nanosheets to form Ir NPs, resulting in the formation of Ir@NiFe-MOF/NF. Importantly, this process is thermodynamically

favorable, as indicated by the positive total potential of Ir and Ni^{2+} generated during the reaction of Ir^{3+} and Ni ($\Delta E = E_{Ir^{III}/Ir^{0}} - E_{Ni^{II}/Ni^{0}} > 0$).

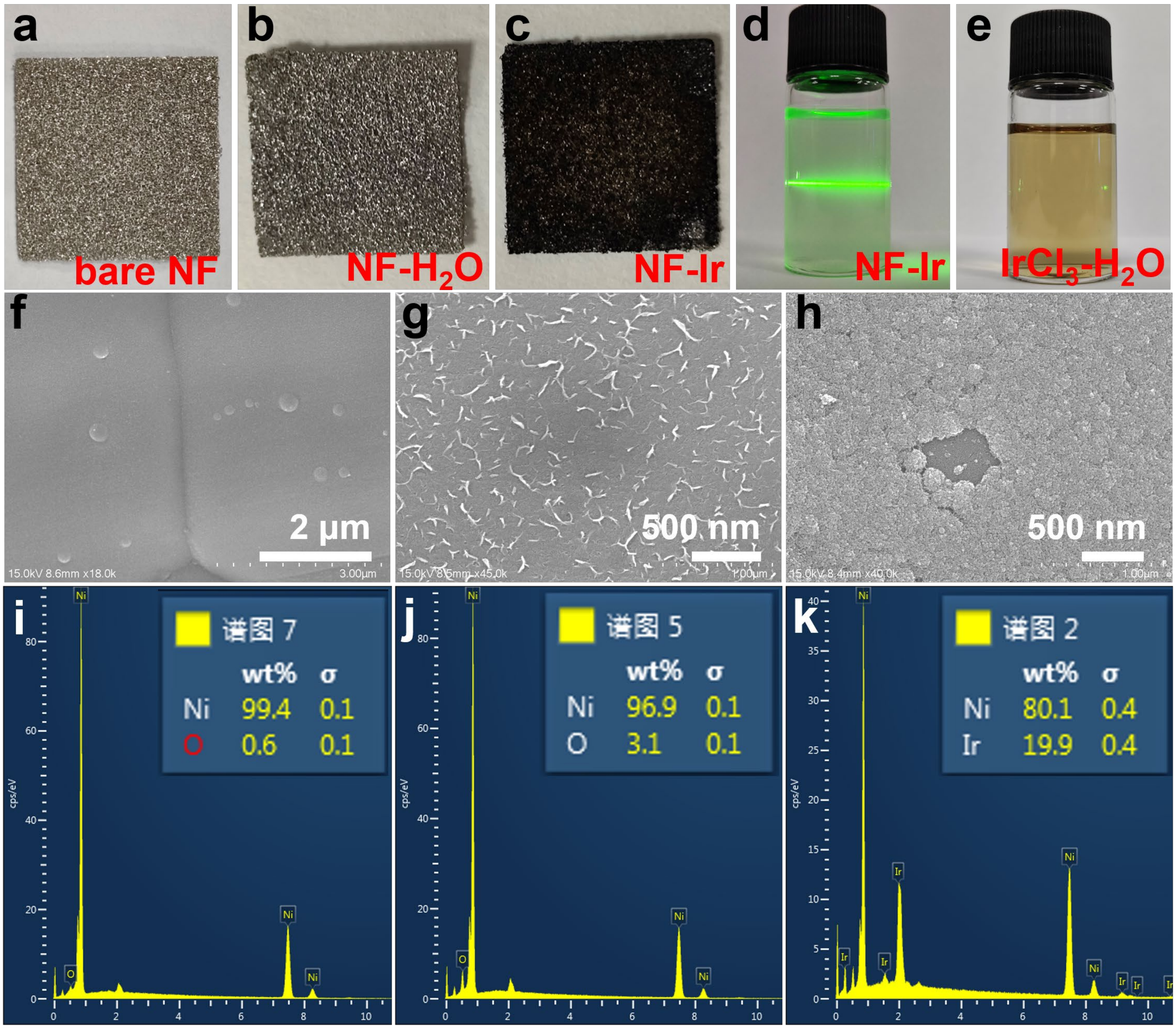

Figure S3. The photographs of (a) bare NF, (b) NF-H_2O, (c) NF-Ir, (d) the solution after the reaction of NF and $IrCl_3$, and (e) the solution after the reaction of NF and H_2O; the SEM images of (f) bare NF, (g) NF-H_2O and (h) NF-Ir; the EDX spectra of (i) bare NF, (j) NF-H_2O and (k) NF-Ir.

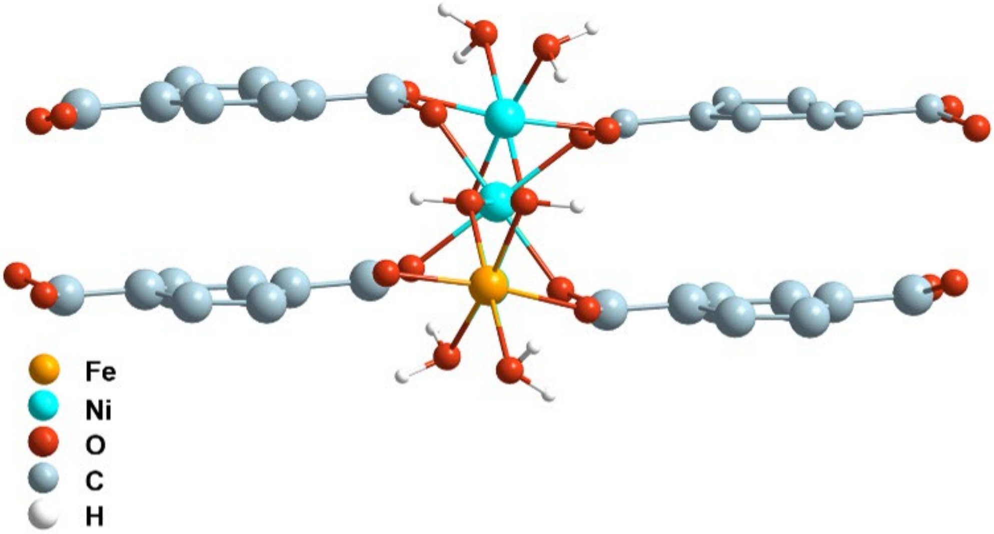

Figure S4. Diagram of the atomic arrangement of NiFe-MOF.

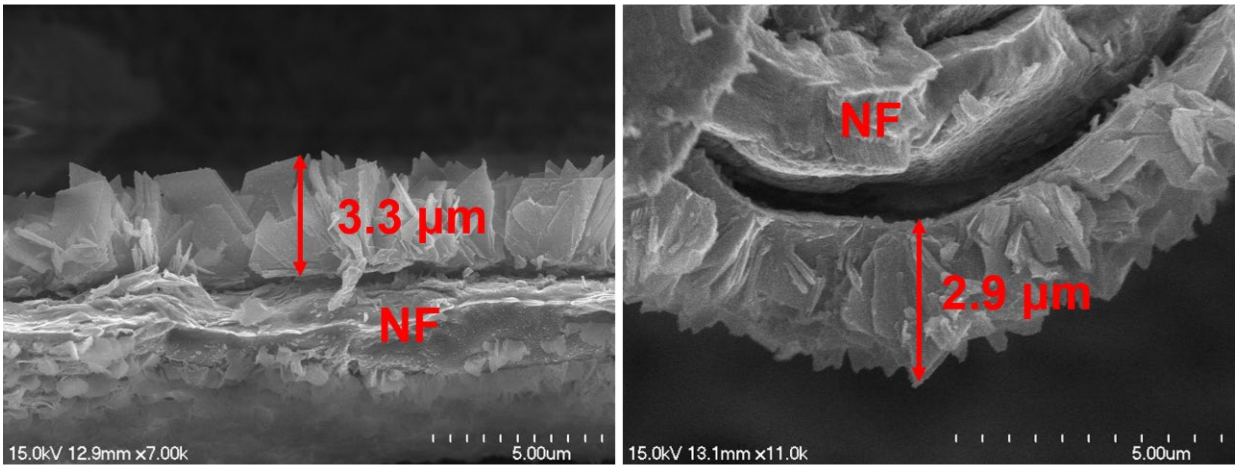

Figure S5. The cross-sectional SEM images of Ir@NiFe-MOF/NF.

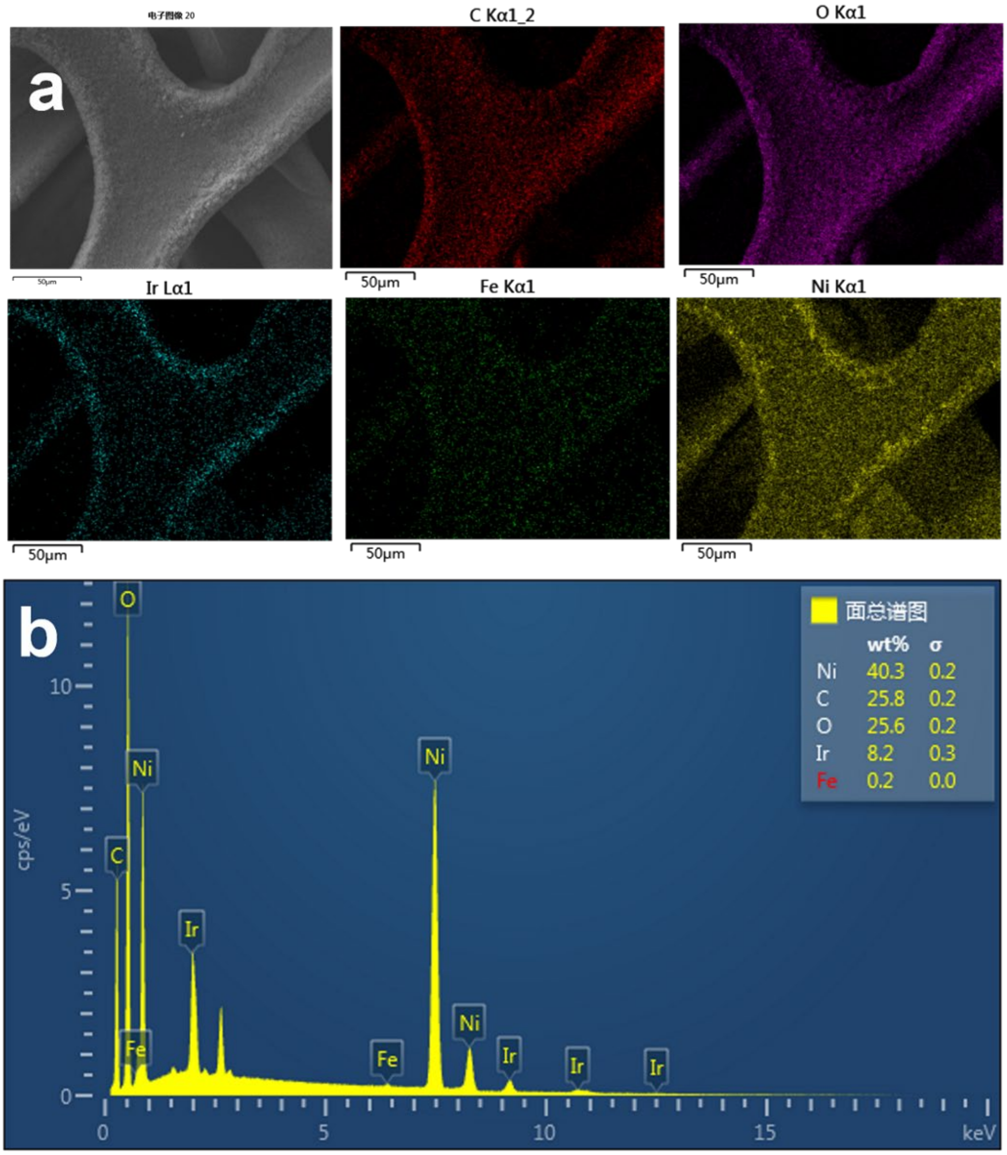

Figure S6. The EDS mapping and the corresponding EDS spectra of Ir@NiFe-MOF/NF.

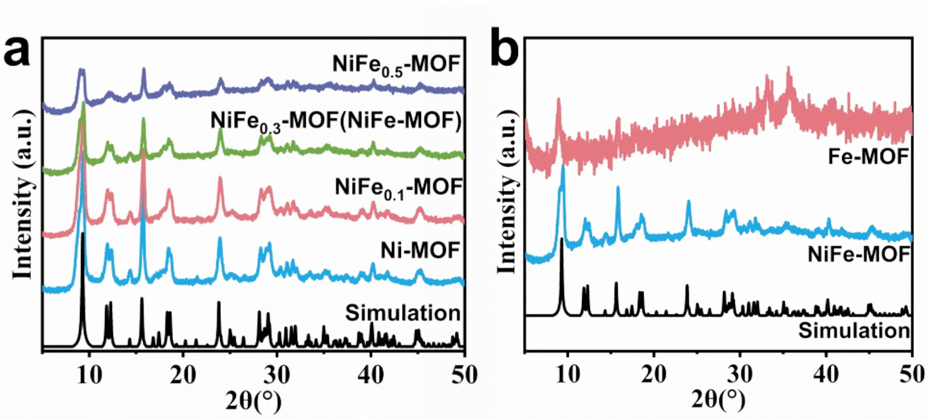

Figure S7. PXRD patterns of (a) Ni-MOF and NiFe-MOF with different amounts of Fe doping, (b) PXRD patterns of Fe-MOF.

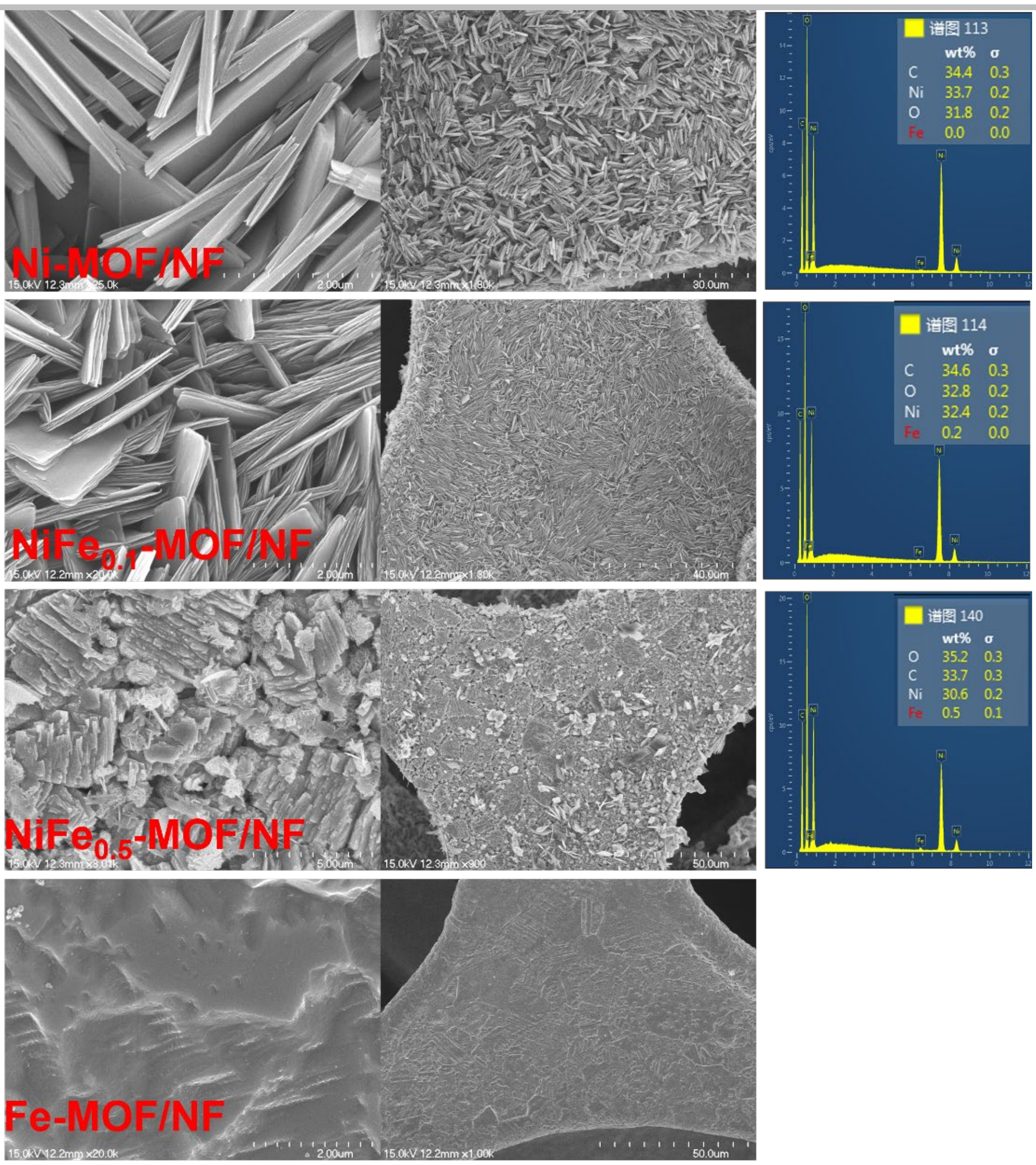

Figure S8. SEM images and EDS spectra of Ni-MOF/NF, $NiFe_{0.1}$-MOF/NF, $NiFe_{0.5}$-MOF/NF and Fe-MOF/NF.

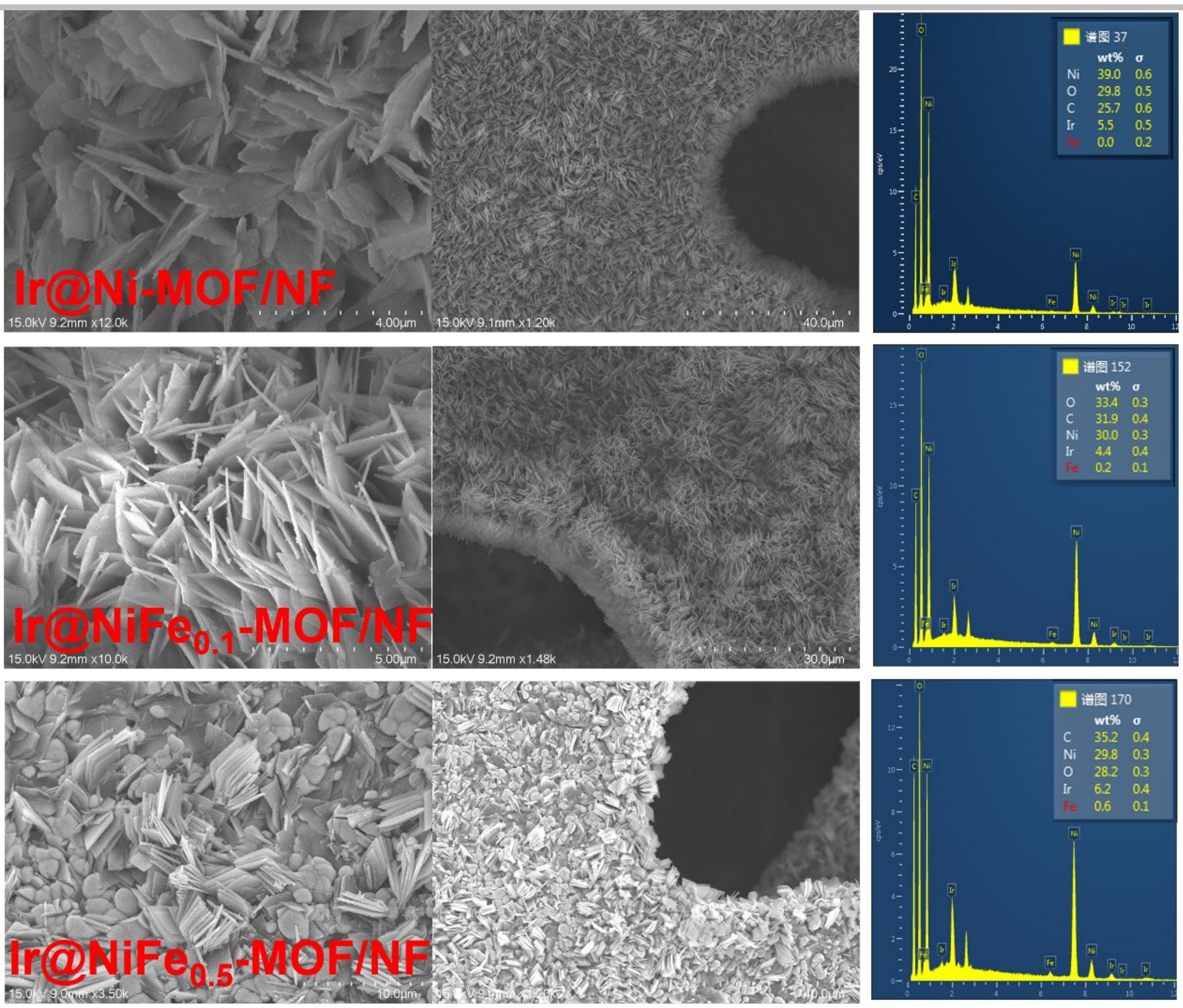

Figure S9. SEM images and EDS spectra of Ir@Ni-MOF/NF, Ir@NiFe0.1-MOF/NF, and Ir@NiFe0.5-MOF/NF.

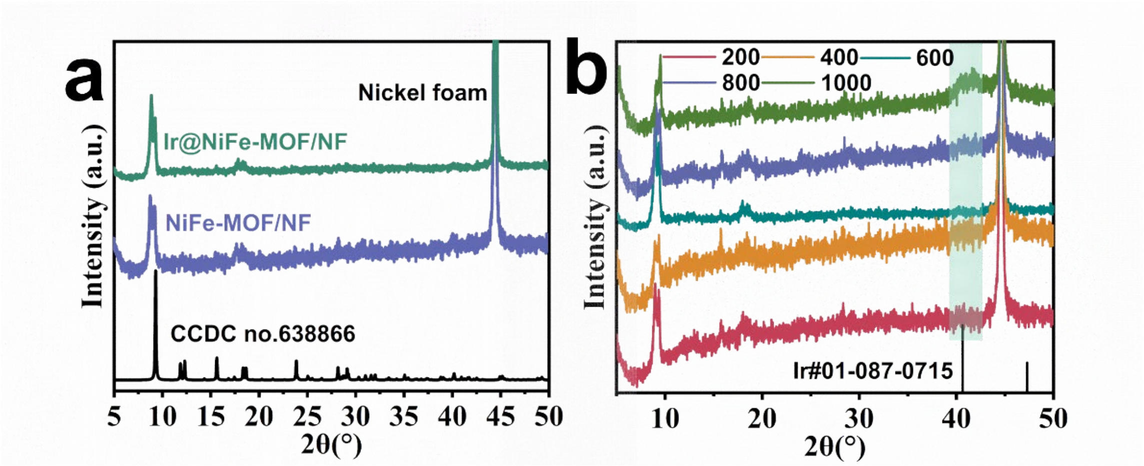

Figure S10. PXRD patterns of (a) NiFe-MOF/NF and Ir@NiFe-MOF/NF and (b) Ir@NiFe-MOF/NF-200/400/600/800/1000.

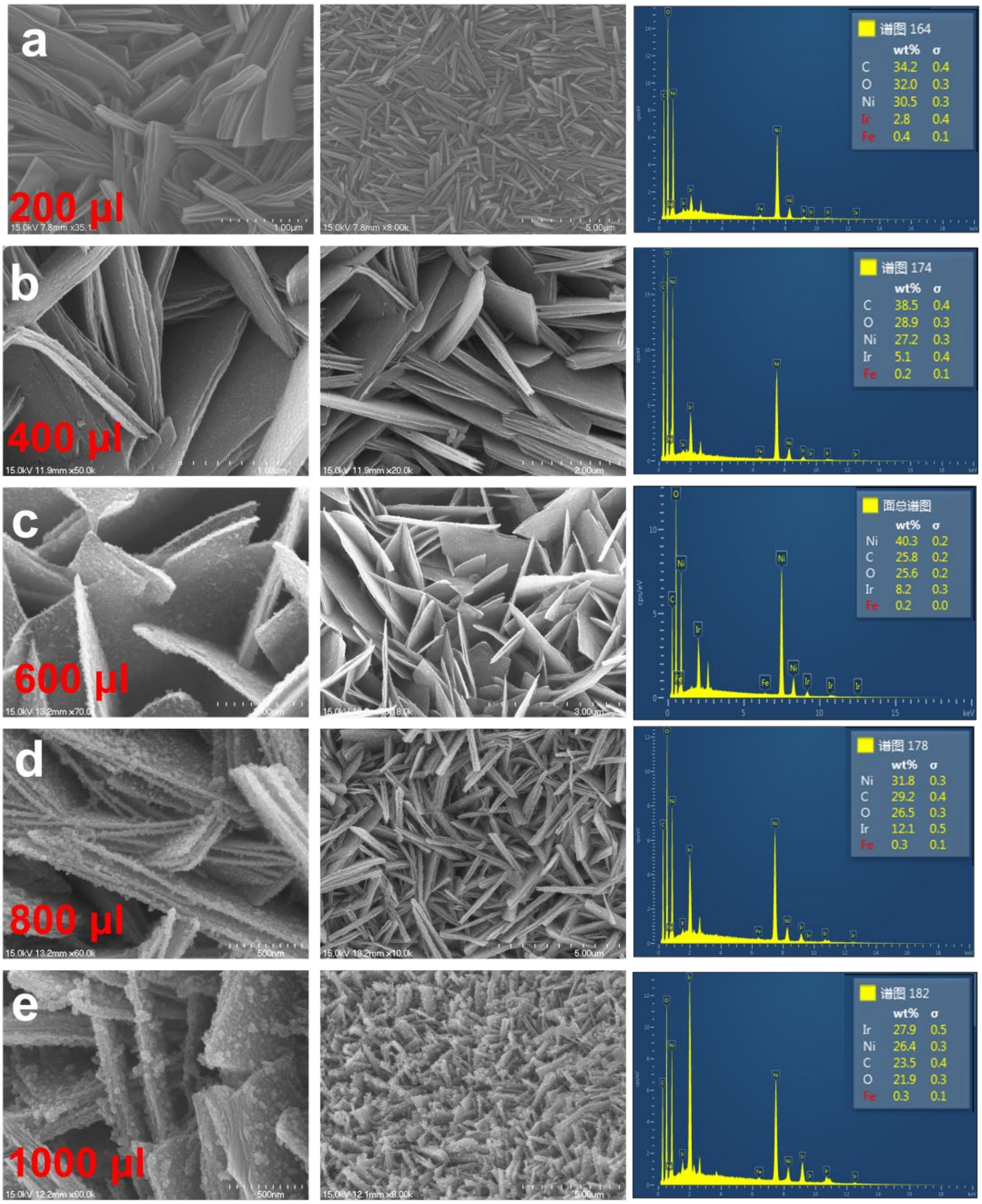

Figure S11. SEM images and EDS spectra of Ir@NiFe-MOF/NF-200/400/600/800/1000.

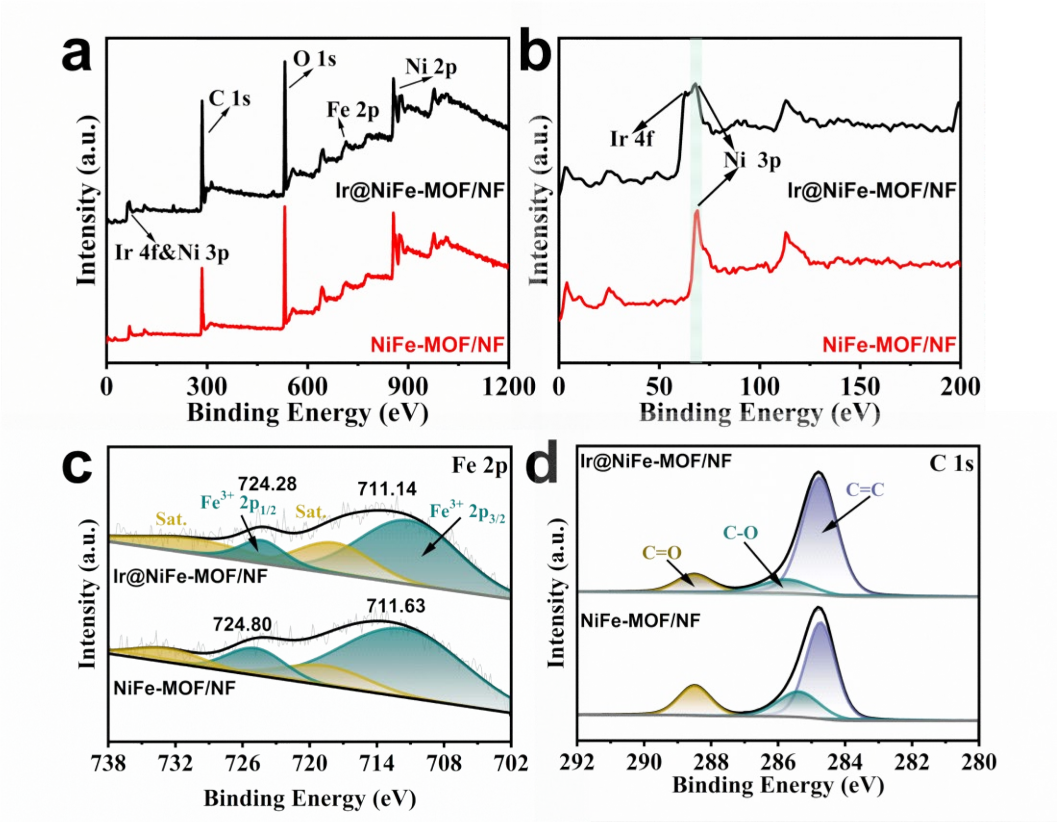

Figure S12. XPS survey spectra (a, b) and High-resolution XPS spectra of the Fe 2p (c) and C 1s (d) of NiFe-MOF/NF and Ir@NiFe-MOF/NF.

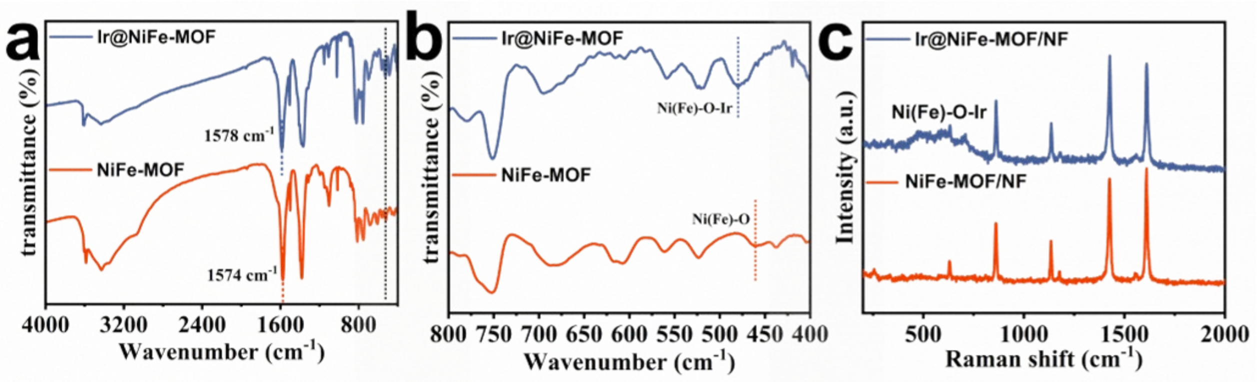

Figure S13. FT-IR spectra (a, b) and Raman spectra (c)of NiFe-MOF/NF and Ir@NiFe-MOF/NF.

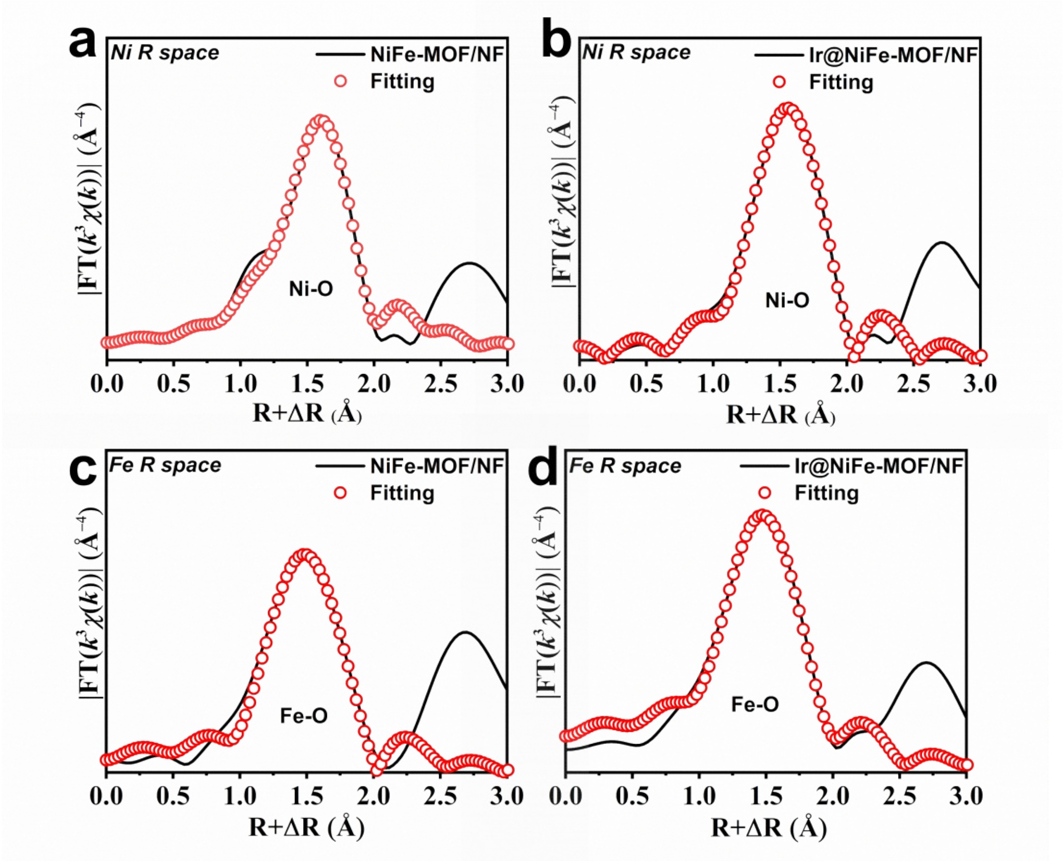

Figure S14. EXAFS fitting spectra of the Ni and Fe K-edge for NiFe-MOF/NF and Ir@NiFe-MOF/NF.

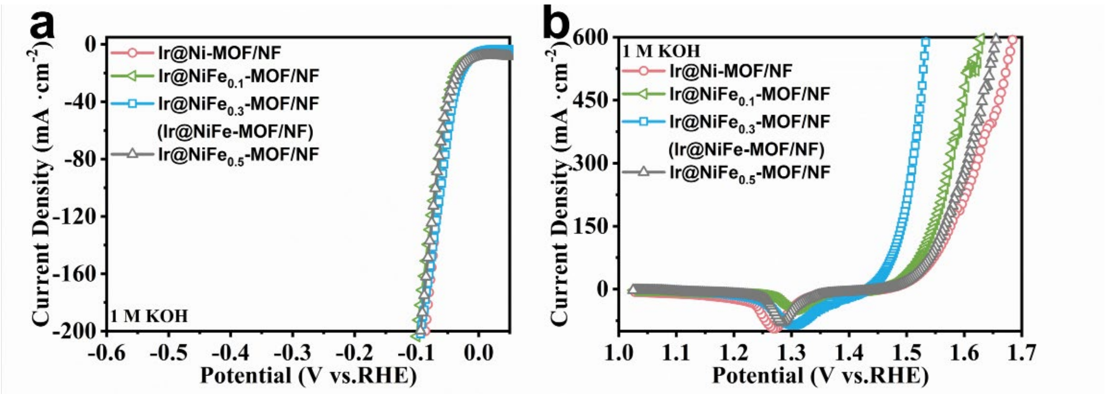

Figure S15. LSV Polarization curves for HER (a) and OER (b) of Ir@Ni-MOF/NF, Ir@NiFe$_{0.1}$-MOF/NF, Ir@NiFe$_{0.3}$-MOF/NF and Ir@NiFe$_{0.5}$-MOF/NF.

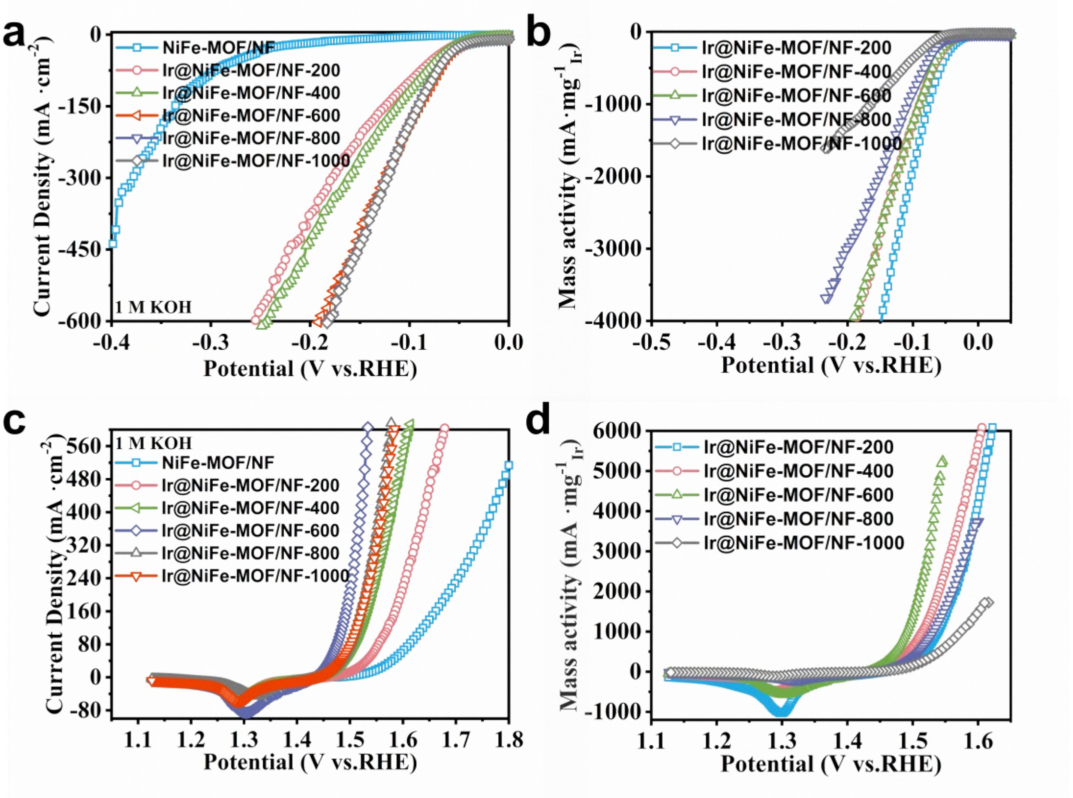

Figure S16. (a) HER polarization curves, (b) the mass-normalized LSV curves, (c) OER polarization curves and (d) the mass-normalized LSV curves of Ir@NiFe-MOF/NF-200/400/600/800/1000 in 1.0 M KOH.

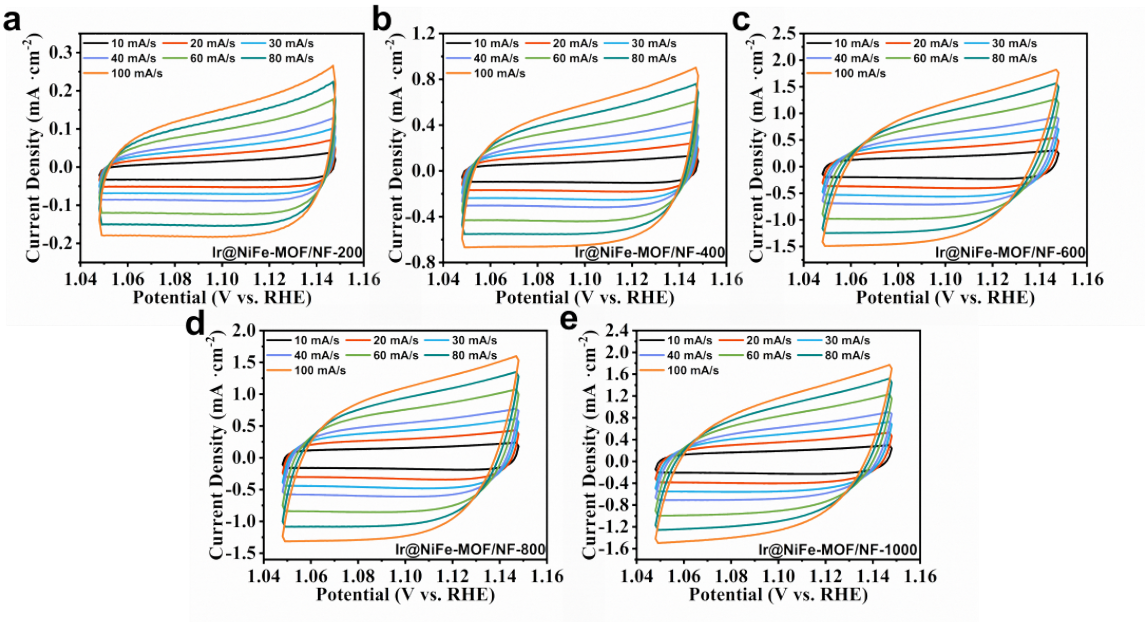

Figure S17. Electrochemical cyclic voltammetry curves for Ir@NiFe-MOF/NF with different Ir content at different scan rates in 1.0 M KOH.

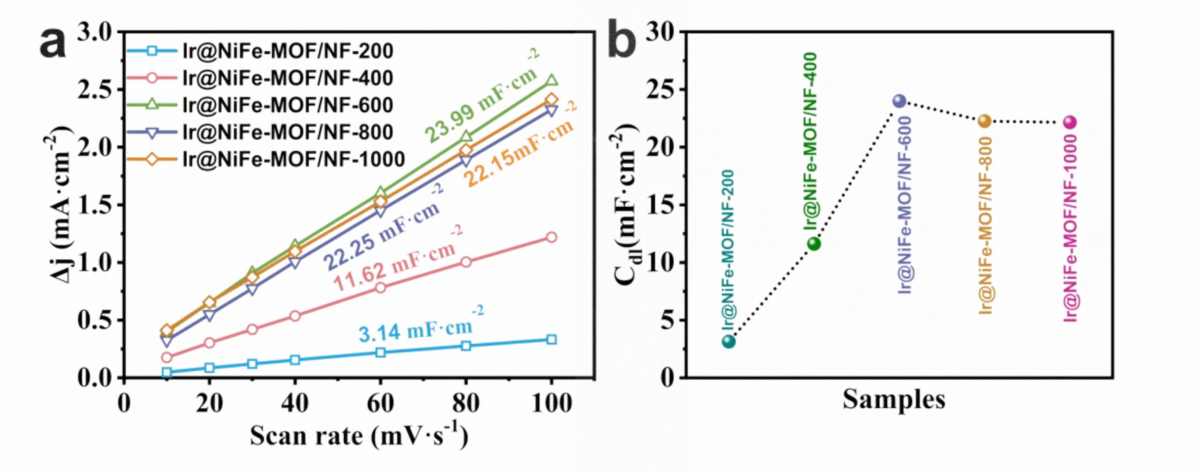

Figure S18. The capacitive currents as a function of the scan rates (a). The double-layer capacitance (C_{dl}) values of different samples (b).

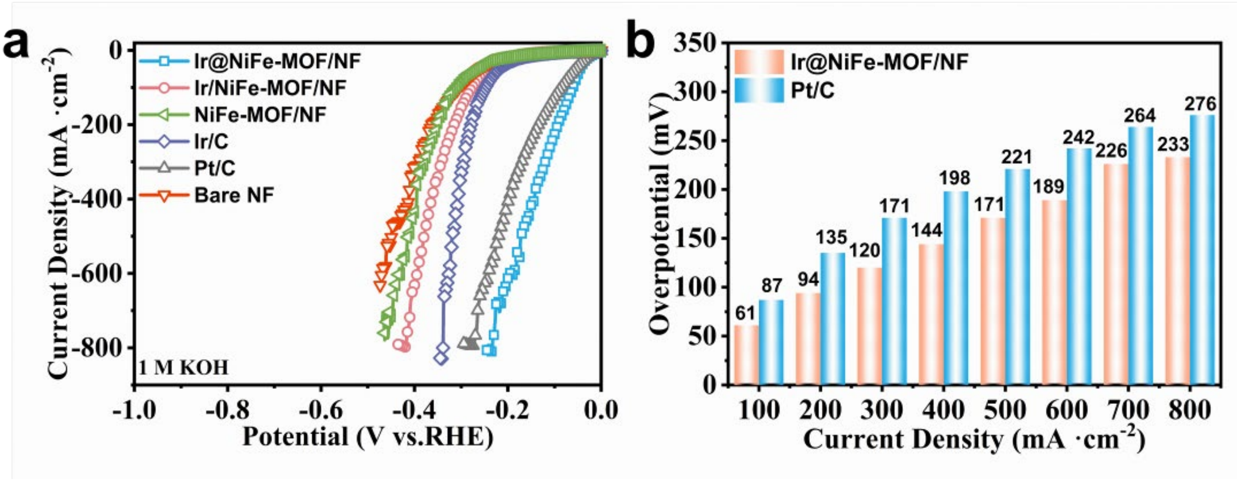

Figure S19. (a) the HER polarization curves of samples in the large-current ranges and (b) the overpotential comparison of Ir@NiFe-MOF/NF and Ir/NiFe-MOF/NF at various current densities.

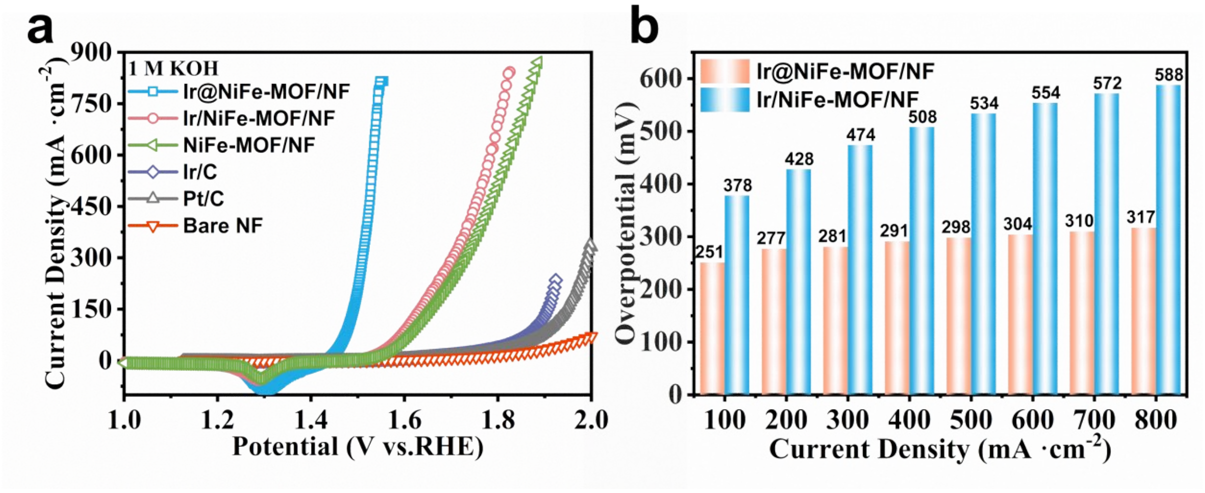

Figure S20. The OER polarization curves of samples in the large-current ranges (a) and the overpotential comparison of Ir@NiFe-MOF/NF and Ir/NiFe-MOF/NF at various current densities (b).

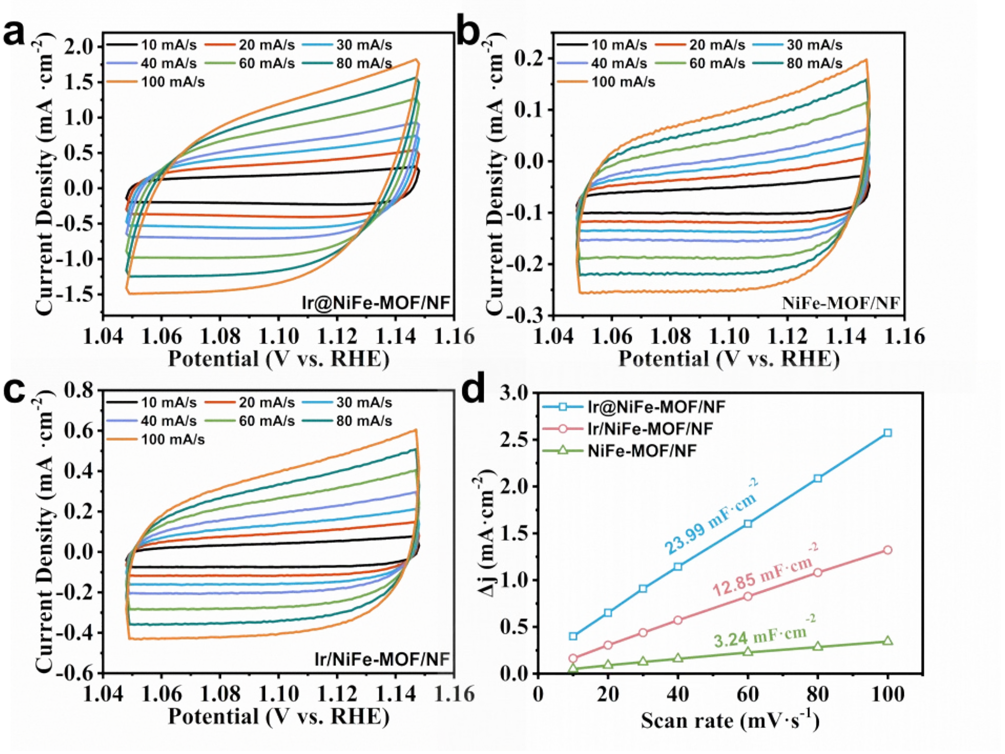

Figure S21. CV curves of (a) Ir@NiFe-MOF/NF, (b) Ir/NiFe-MOF/NF, and (c) NiFe-MOF/NF at increasing scan rates from 10-100 mA·s^{-1} in 1.0 M KOH and (d)The double-layer capacitance (C_{dl}) values of different samples.

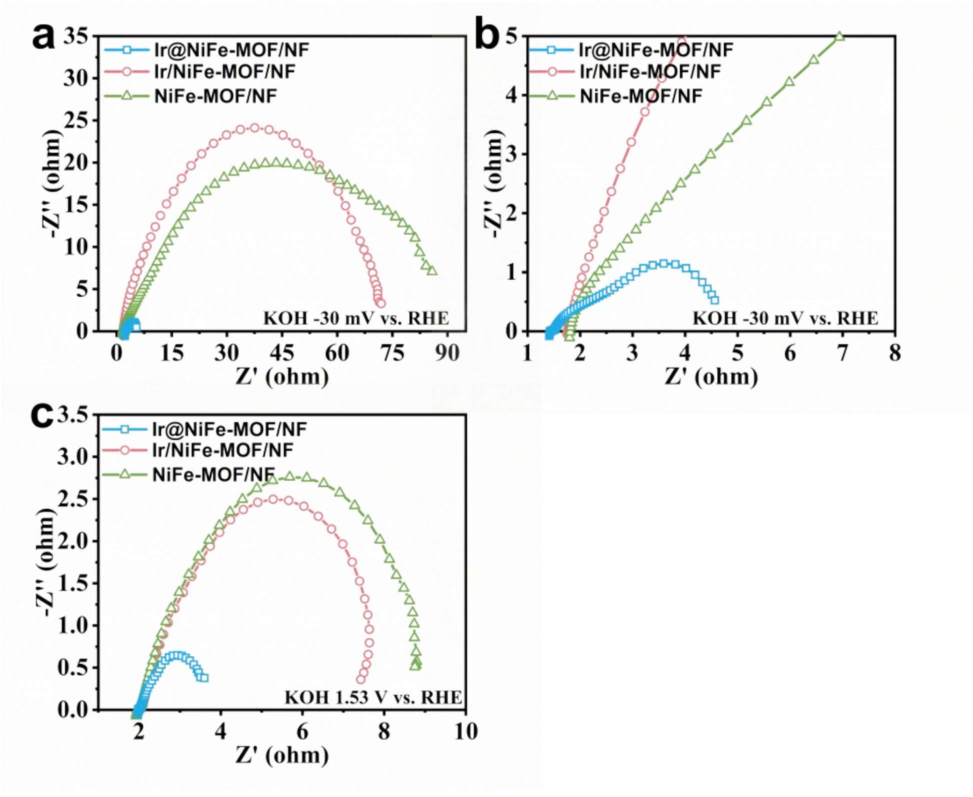

Figure S22. Nyquist plots measured at the potential of (a, b) -30 mV for HER and (c) 1.53 V for OER in 1.0 M KOH.

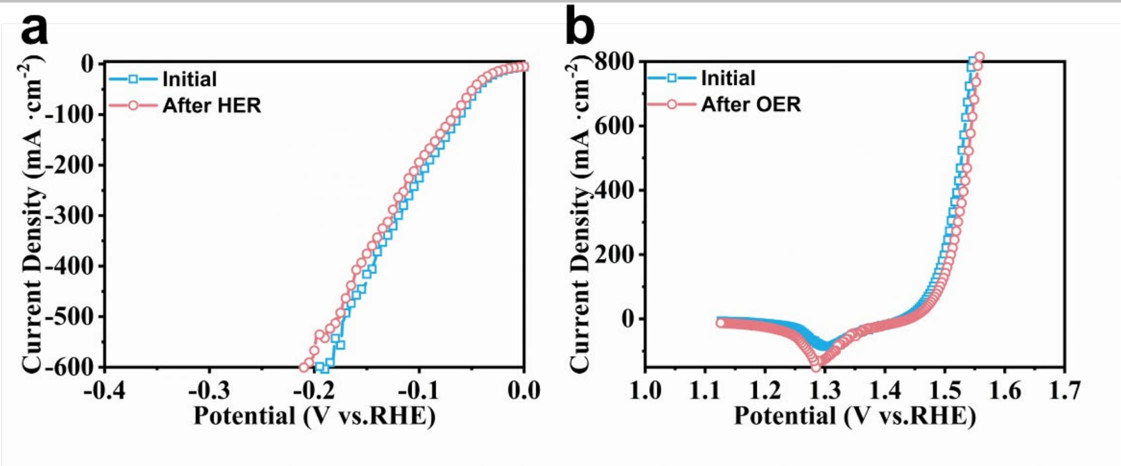

Figure S23. The LSV curves of Ir@NiFe-MOF/NF before and after (a) HER and (b) OER tests.

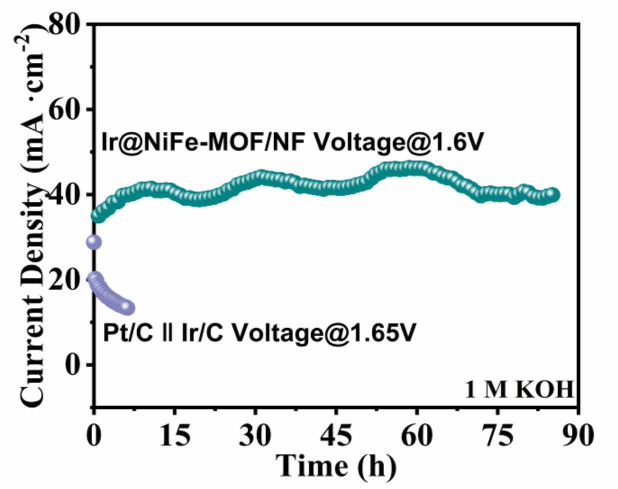

Figure S24. The i-t curves of Ir@NiFe-MOF/NF with 1.6 V toward overall water splitting.

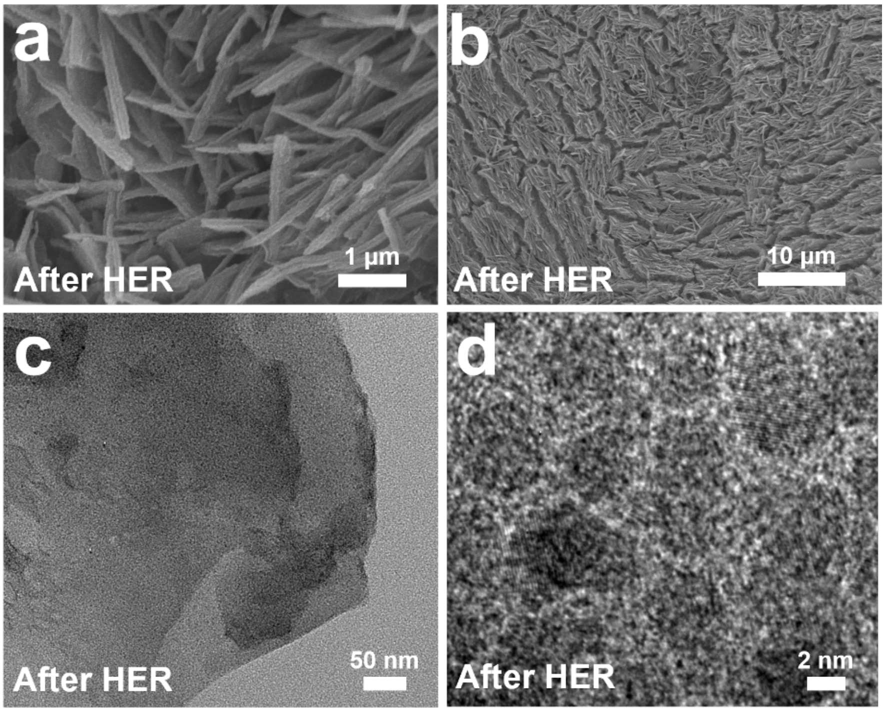

Figure S25. SEM (a, b) and TEM (c, d) images of Ir@NiFe-MOF/NF after HER in 1 M KOH.

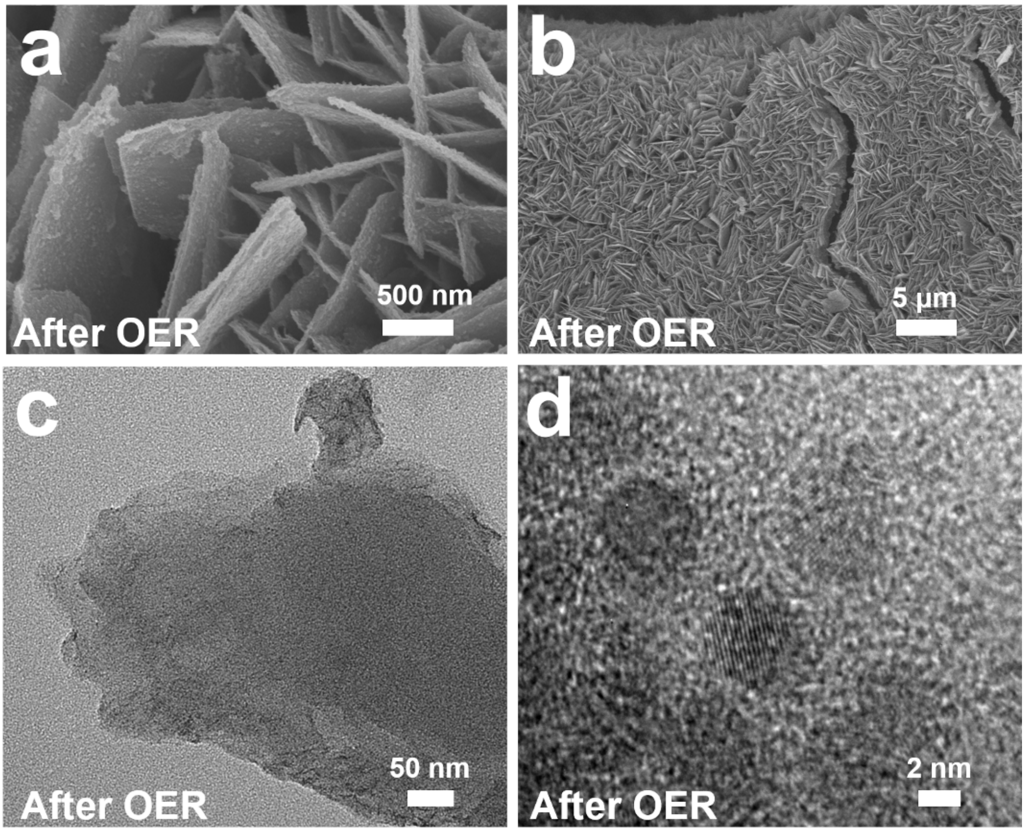

Figure S26. SEM (a, b) and TEM (c, d) images of Ir@NiFe-MOF/NF after OER in 1 M KOH.

Noted: The result showed no significant changes in the microstructure of the title catalyst.

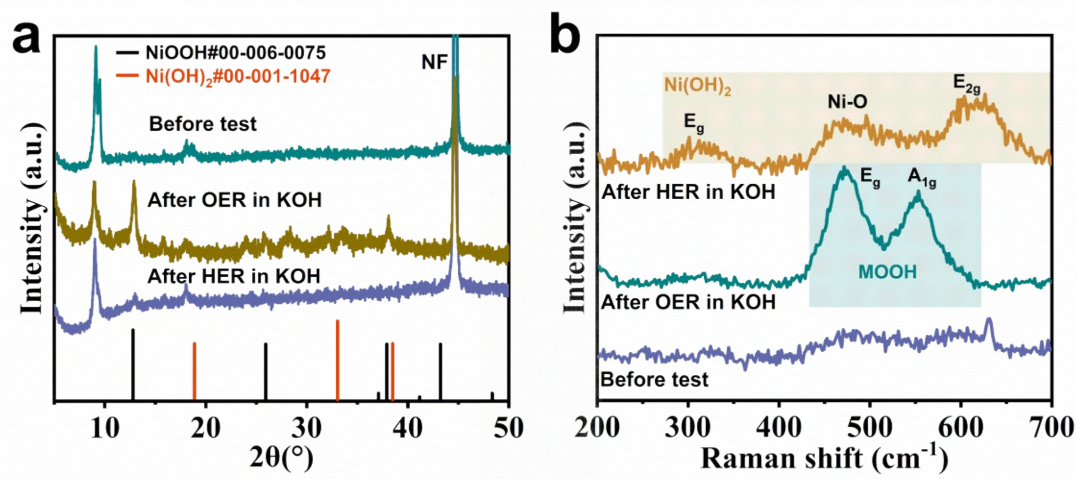

Figure S27. PXRD patterns (a) and Raman spectra (b) of Ir@NiFe-MOF/NF after HER and OER in 1 M KOH.

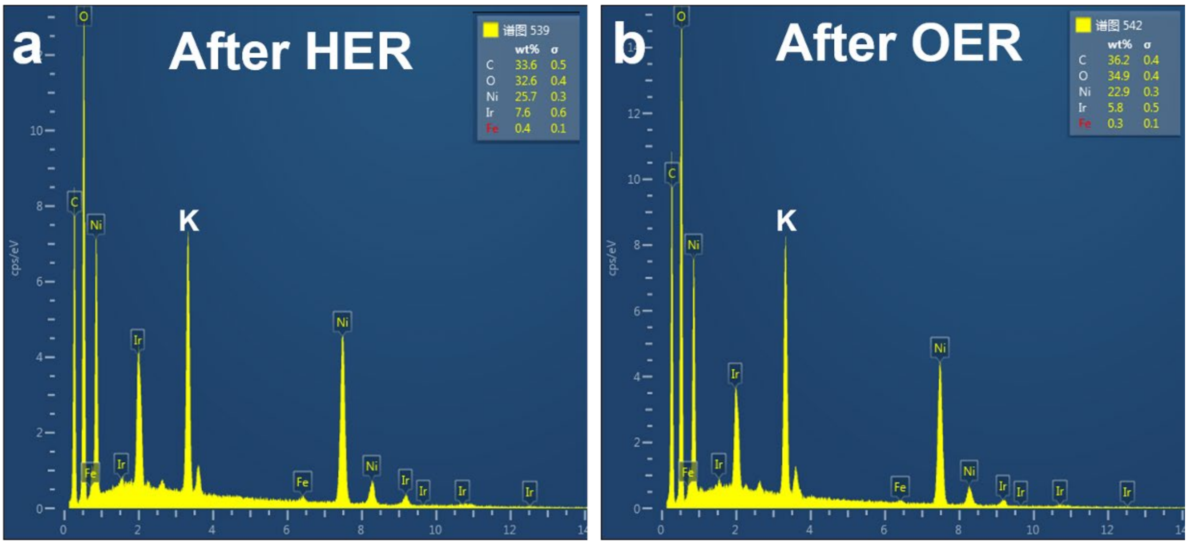

Figure S28. The EDS spectra of Ir@NiFe-MOF/NF after (a) HER and (b) OER in 1 M KOH.

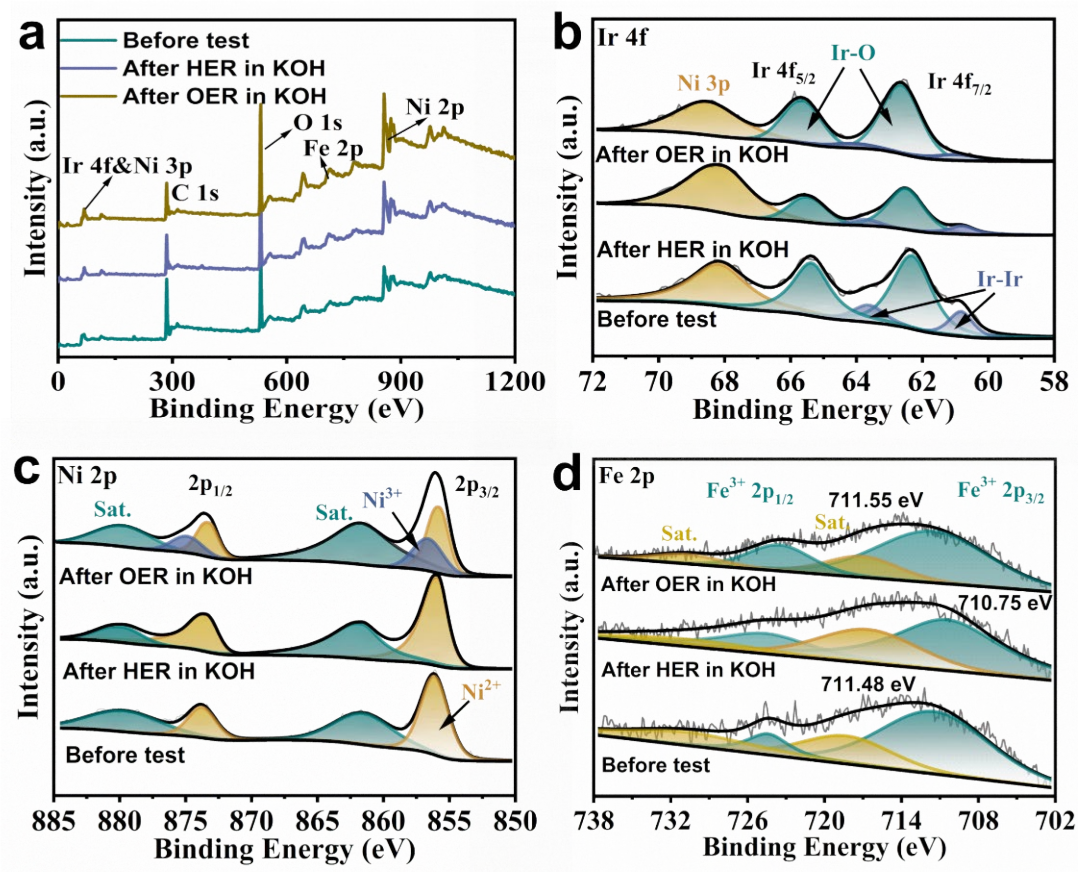

Figure S29. (a) XPS survey spectra and High-resolution XPS spectra of Ir 4f (b), Ni 2p (c), and Fe 2p (d) of Ir@NiFe-MOF/NF after HER and OER in 1 M KOH.

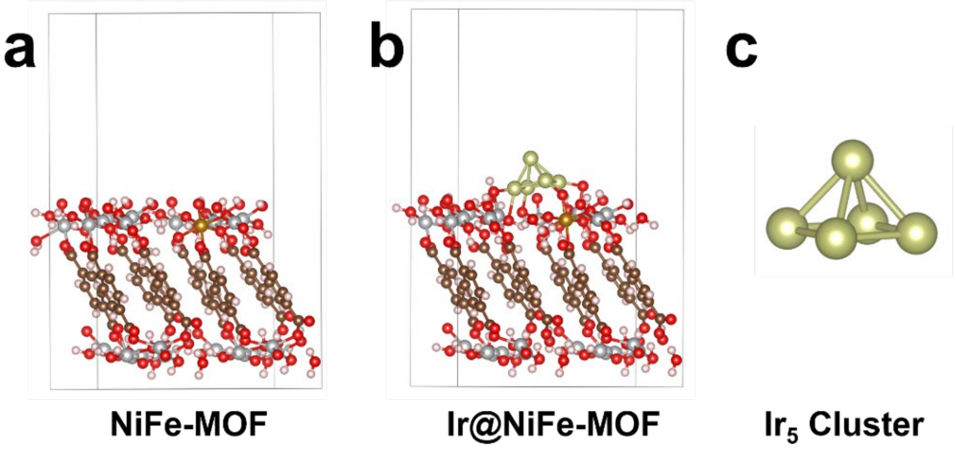

Figure S30. Structural models of (a) NiFe-MOF and (b) Ir@NiFe-MOF and (c) Ir NPs.

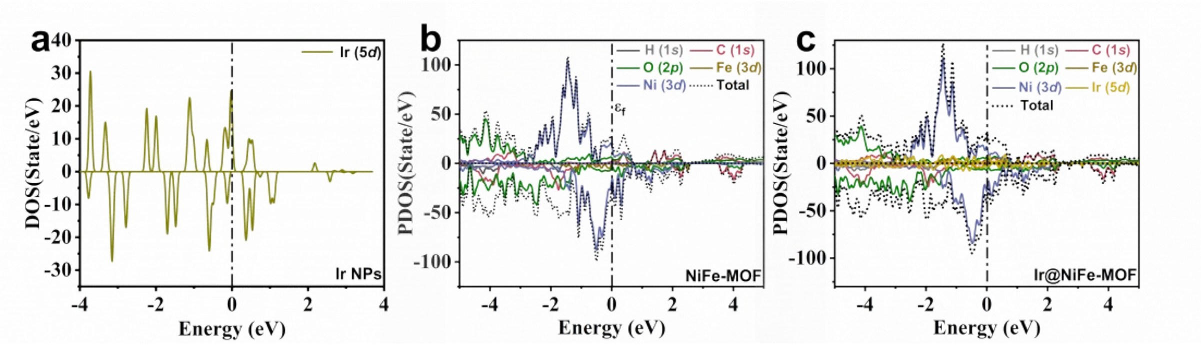

Figure S31. The DOS plots for Ir NPs, NiFe-MOF and Ir@NiFe-MOF. The dashed line indicates the Fermi level for each system.

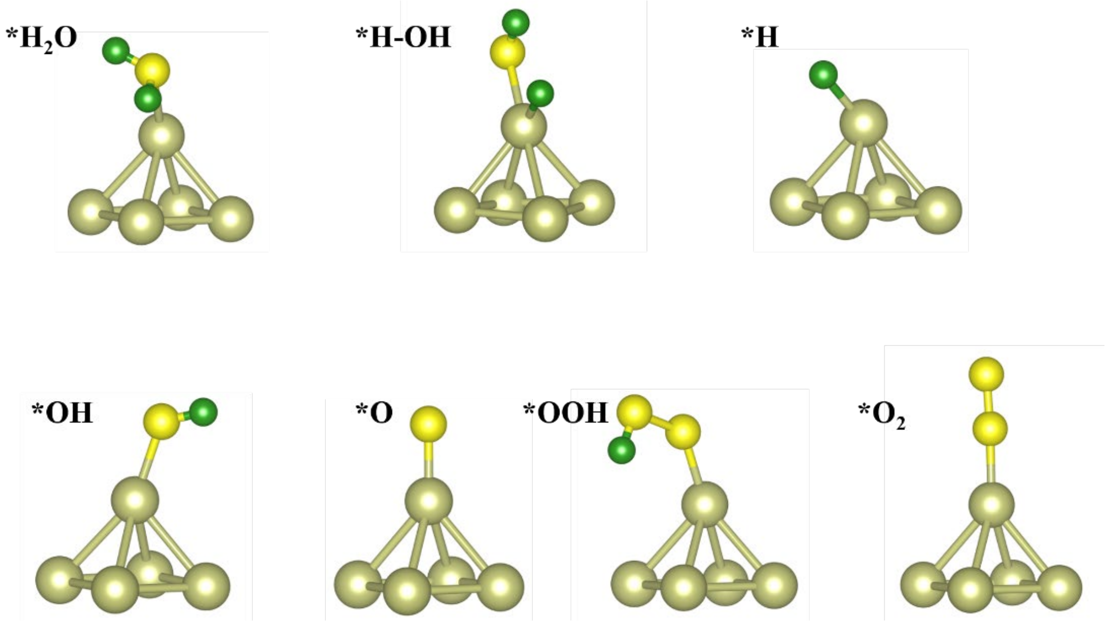

Figure S32. The optimized adsorption structures of HER and OER process intermediates at Ir NPs.

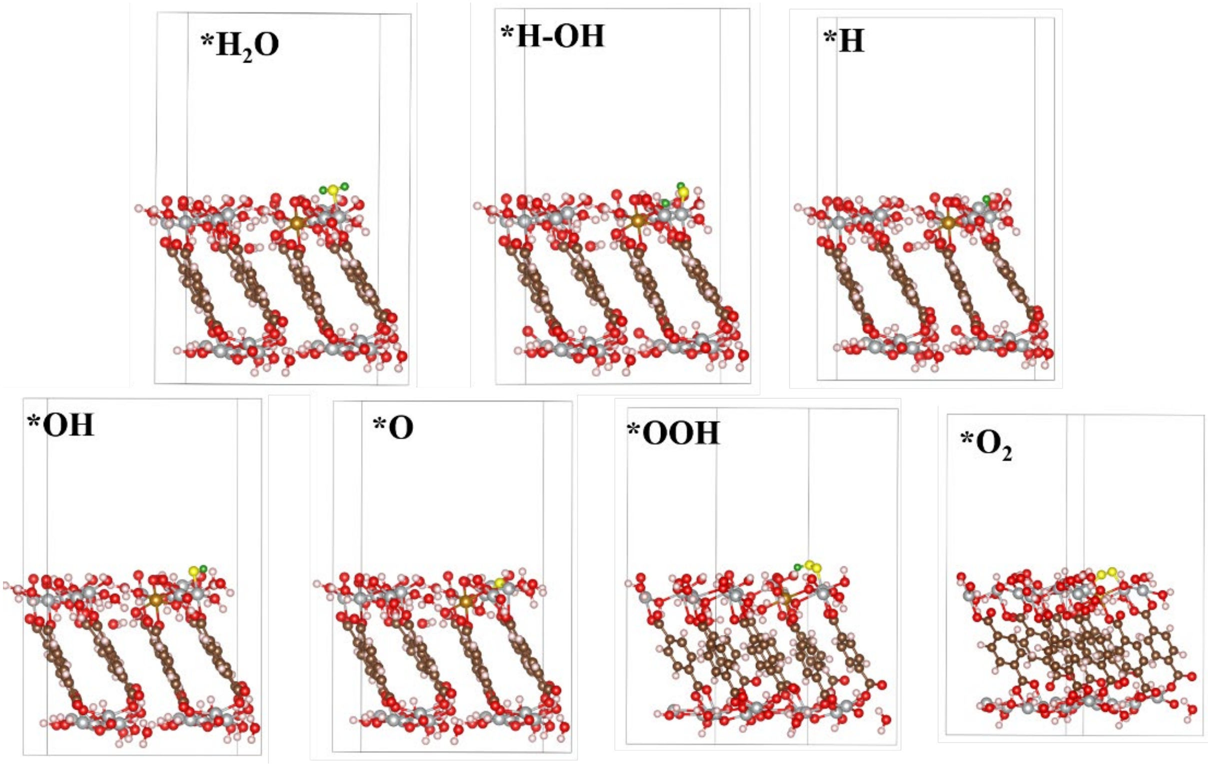

Figure S33. The optimized adsorption structures of HER and OER process intermediates at NiFe-MOF.

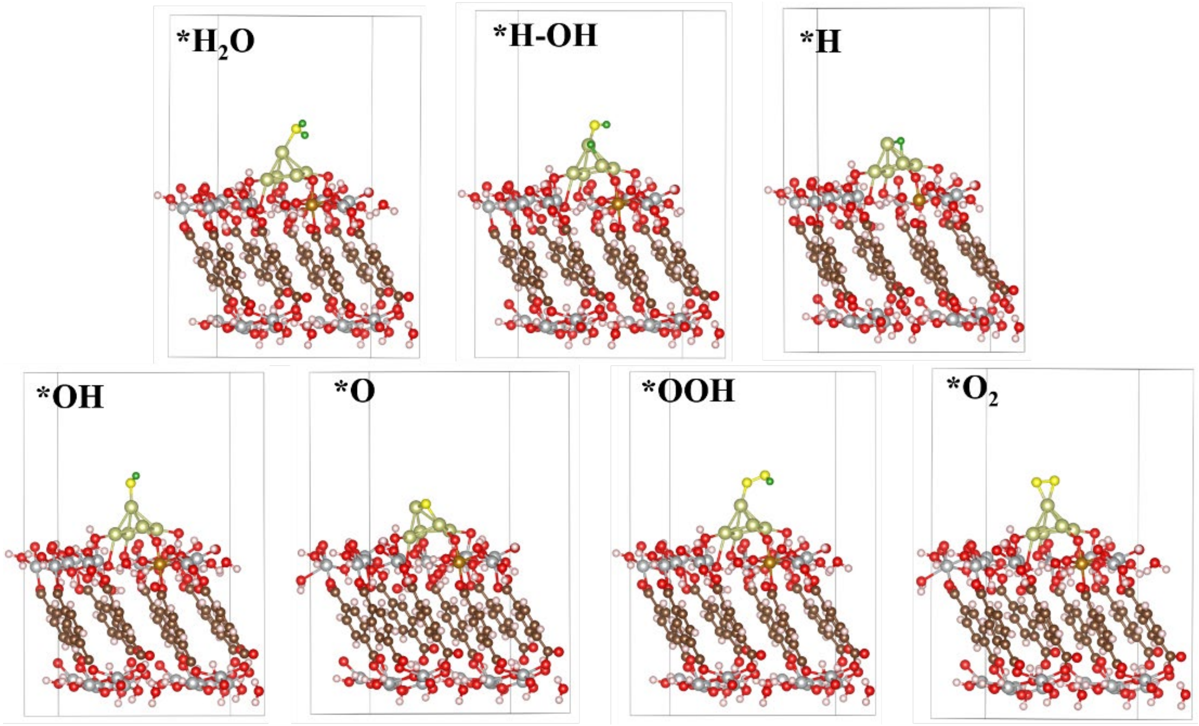

Figure S34. The optimized adsorption structures of HER and OER process intermediates at Ir@NiFe-MOF.

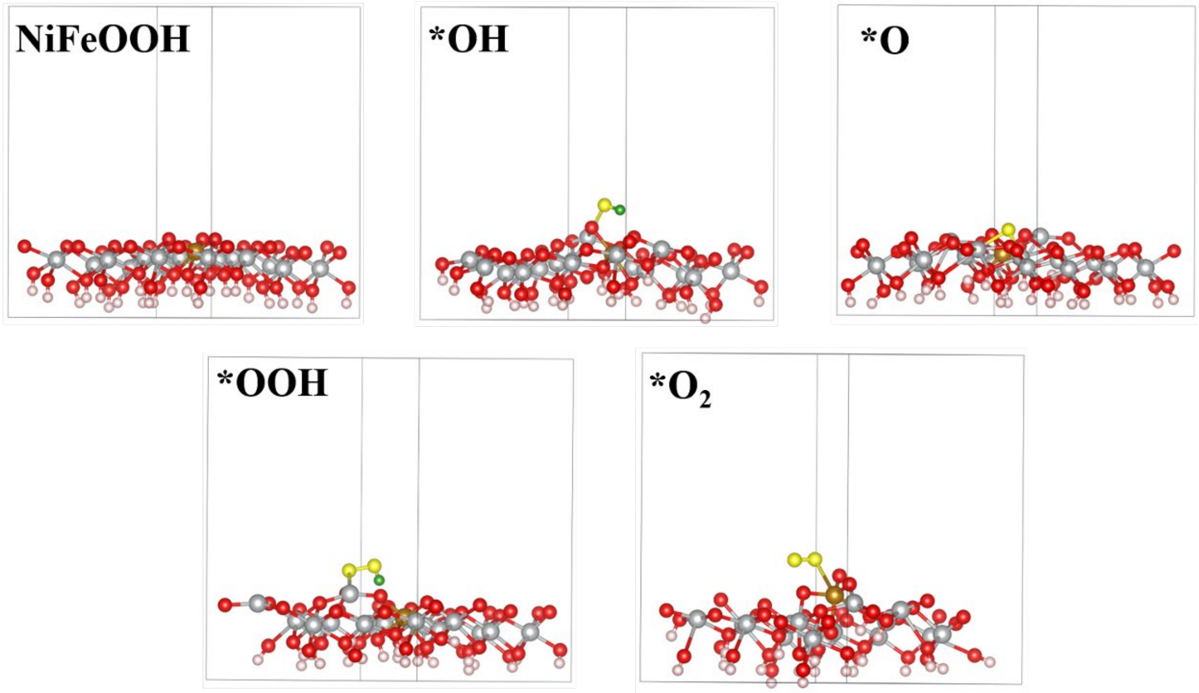

Figure S35. The optimized adsorption structures of OER process intermediates at NiFeOOH.

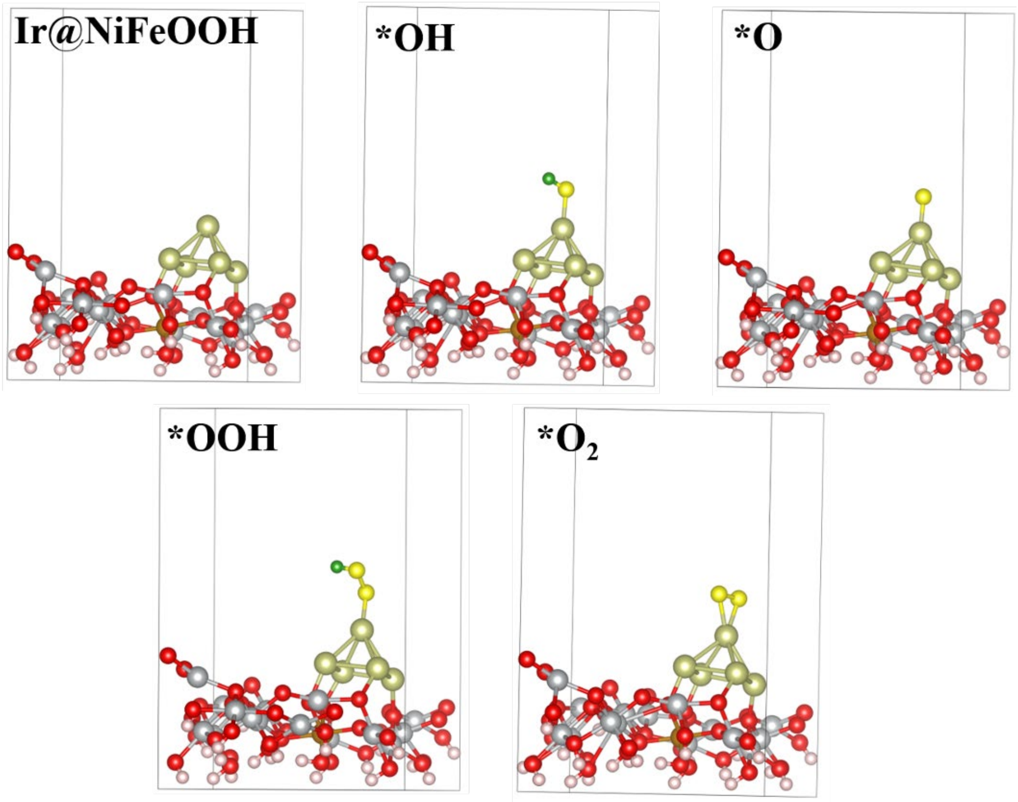

Figure S36. The optimized adsorption structures of OER process intermediates at Ir@NiFeOOH.

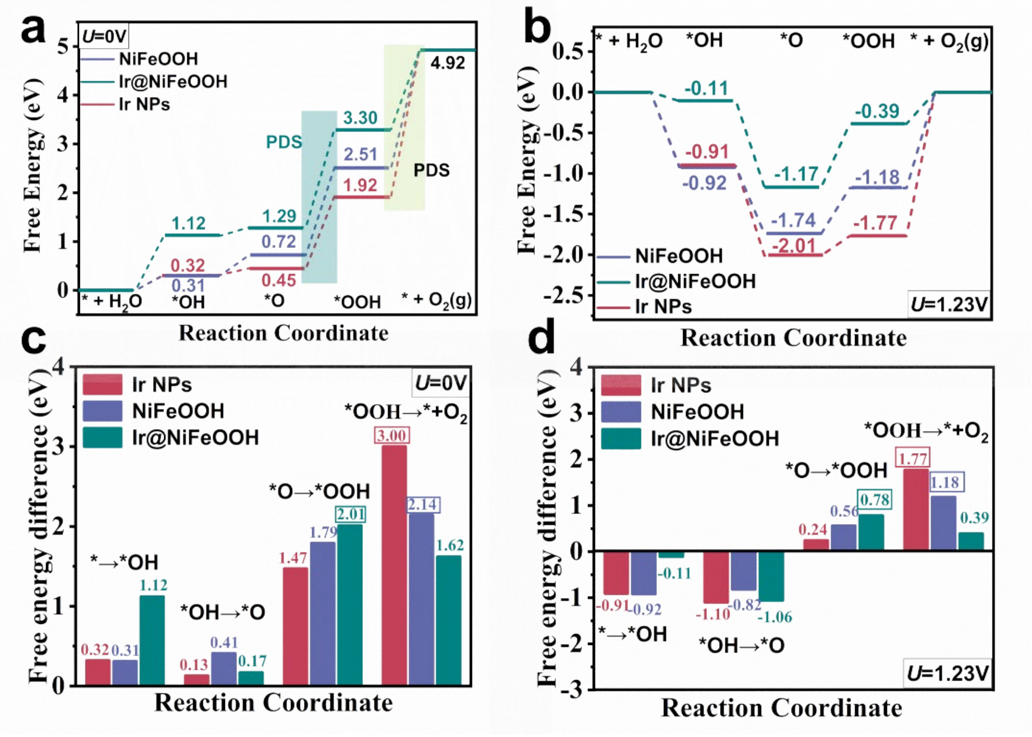

Figure S37. (a, b) Reaction pathways in the OER process, and (c, d) corresponding free energy difference for Ir NPs, NiFeOOH and Ir@NiFeOOH.

3. Supplementary Tables

Table S1. The compositions of the pristine NiFe-MOF/NF and Ir@NiFe-MOF/NF with different amounts of $IrCl_3$ determined by ICP-OES.

Catalyst	Ni (wt.%)	Fe (wt.%)	Ir (wt.%)
**NiFe-MOF/NF**	33.74	0.36	-
**Ir@NiFe-MOF/NF-200**	29.18	0.38	2.67
**Ir@NiFe-MOF/NF-400**	29.05	0.33	5.08
**Ir@NiFe-MOF/NF**	32.48	0.35	8.14
**Ir@NiFe-MOF/NF-800**	29.94	0.28	12.03
**Ir@NiFe-MOF/NF-1000**	28.65	0.32	27.51

Table S2. The weight of Ir@NiFe-MOF can be estimated by measuring the difference in mass after stripping it off from the NF through high-power sonication. The area of Ir@NiFe-MOF/NF is 2 cm × 2 cm.

$Weight_0$ (mg)	$Weight_1$ (mg)	Loss weight (mg)	Weight density (mg/cm^{-2})
**117.0**	109.4	7.6	1.9
**121.9**	114.7	7.2	1.8
**116.5**	109.2	7.3	1.83
**123.1**	115.3	7.8	1.95

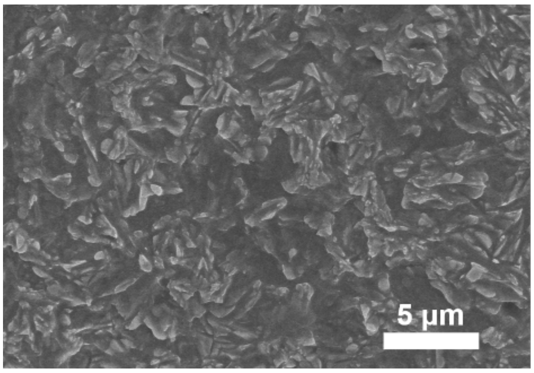

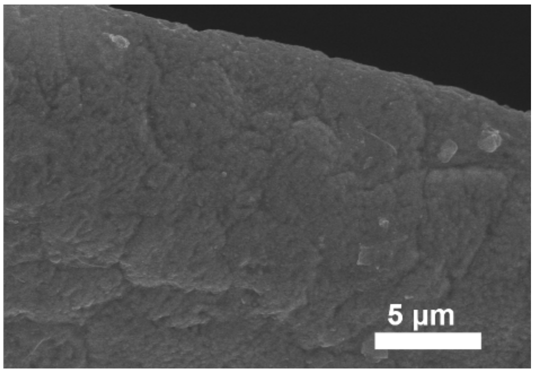

Noted: The above SEM images shown that the Ir@NiFe-MOF was almost completely detached from the nickel foam by high-power sonication of the Ir@NiFe-MOF/MOF electrode.

Table S3. EXAFS fitting parameters at the Ni and Fe K-edge for NiFe-MOF/NF and Ir@NiFe-MOF/NF.

Sample	Shell	N^a	$R(Å)^b$	$\sigma^2(Å^2)^c$	$\Delta E_0(eV)^d$	*R factor*
**NiFe-MOF/NF**	Ni-O	5.4	2.06	0.0053	0.613	0.0050
**Ir@NiFe-MOF/NF**	Ni-O	6.2	2.05	0.0078	0.482	0.0008
**NiFe-MOF/NF**	Fe-O	5.3	2.00	0.0085	-1.371	0.0037
**Ir@NiFe-MOF/NF**	Fe-O	5.6	1.99	0.0067	0.0460	0.0021

^aN: coordination numbers (CN); ^bR: bond distance; $^c\sigma^2$: Debye-Waller factors; $^d\Delta E_0$: the inner potential correction. *R* factor: goodness of fit. S_0^2 (amplitude decay factor) was set to 1.0326 for Ni and 0.9380 for Fe, according to the experimental EXAFS fit of Ni foil and Fe foil by fixing CN as the known crystallographic value.

Table S4. Comparison of Ir@NiFe-MOF/NF with the reported electrocatalysts for HER performance in alkaline.

Electrocatalysts	η_{10} (mV)	Tafel slope (mV dec^{-1})	Electrolyte	References
**Ir@NiFe-MOF/NF**	**15**	**43.2**	**1 M KOH**	**This work**
Ir@Ni-NDC	19	28.2	1 M KOH	*Angew. Chem. Int. Ed.* **2023**, *62*, e202302220.
Ru@Ni-MOF	22	40	1 M KOH	*Angew. Chem. Int. Ed.* **2021**, *60*, 22276.
$NiRu_{0.13}$-BDC	34	32	1 M KOH	*Nat. Commun.* **2021**, *12*, 1369.
Ir-NR/C	42	35.2	1 M KOH	*Appl. Catal. B Environ.* **2020**, *279*, 119394.
IrO_2/V_2O_5	49	48	1 M KOH	*Adv. Sci.* **2022**, *9*, 2104636.
IrCo@NC	82	56	1 M KOH	*Adv. Funct. Mater.* **2021**, *31*, 2101797.
Pt-NC/Ni-MOF	25	42.1	1 M KOH	*Chem* **2019**, *5*, 2429.
NiRu-MOF/NF	51	90	1 M KOH	*ACS Appl. Mater. Interf.* **2020**, *12*, 34728.
Ru/Co-CAT/CC	38	32.1	1 M KOH	*Adv. Energy Mater.* **2023**, *13*, 2204177.
Ru@Ni-MOF/NF	25	31	1 M KOH	*Chem. Eng. J.* **2023**, *451*, 138618.
2D MOF@Pt	102	52	1 M KOH	*Nano Lett.* **2019**, *19*, 8447−8453.
Pt/MOF-O	66	101.6	1 M KOH	*J. Am. Chem. Soc.* **2021**, *143*, 16512−16518.
IrFe/NC	22	30	1 M KOH	*Appl. Catal. B Environ.* **2019**, *258*, 117965.
Pt@LDH-4h	58	43	1 M KOH	*Small* **2023**, *19*, 2207044.
MIL-(IrNiFe)@NF	12	53	1 M KOH	*J. Mater. Chem. A* **2021**, *9*, 27424–27433
Fe−Co−Ni MOF	116	56	1.0 M KOH	*J. Am. Chem. Soc.* **2022**, *144*, 3411.
NiFe-MOF/NF	240	/	0.1 M KOH	*Nat. Commun.* **2017**, *8*, 15341.

Table S5. Comparison of Ir@NiFe-MOF/NF with the reported electrocatalysts for OER performance in alkaline.

**Electrocatalysts**	**η_{10} (mV)**	**Tafel slope (mV dec^{-1})**	**Electrolyte**	**References**
**Ir@NiFe-MOF/NF**	**213**	**38.5**	**1 M KOH**	**This work**
Ir-NR/C	296	60.3	1 M KOH	*Appl. Catal. B Environ.* **2020**, *279*, 119394.
IrO_2/V_2O_5	283	34	1 M KOH	*Adv. Sci.* **2022**, *9*, 2104636.
IrCo@NC	302	52	1 M KOH	*Adv. Funct. Mater.* **2021**, *31*, 2101797.
Ir-NSs	266	29.1	1 M KOH	*Natl. Sci. Rev.* **2020**, *7*, 1340.
Pt-NC/Ni-MOF	292	/	1 M KOH	*Chem* **2019**, *5*, 2429.
Ru/Co-CAT/CC	201	45.7	1 M KOH	*Adv. Energy Mater.* **2023**, *13*, 2204177.
d-ZnIr$(OH)_6$ NSs/C	231	35.4	1 M KOH	*Energy Environ. Sci.* **2022**, *15*, 1672.
Ir–NiCo LDH	192	41.2	1 M KOH	*J. Mater. Chem. A* **2020**, *8*, 9871.
Fe−Co−Ni MOF	254	51.3	1 M KOH	*J. Am. Chem. Soc.* **2022**, *144*, 3411.
Pt@LDH-4h	239	28.4	1 M KOH	*Small* **2023**, *19*, 2207044.
c/a-NiFe-MOF	236	30	1 M KOH	*Chem. Commun.* **2022**, *58*, 6833.
MIL-(IrNiFe)@NF	η_{50} =230	60	1 M KOH	*J. Mater. Chem. A* **2021**, *9*, 27424–27433
NiFe-MOF/NF	240	34	0.1 M KOH	*Nat. Commun.* **2017**, *8*, 15341.

Table S6. Comparison of Ir@NiFe-MOF/NF with the reported electrocatalysts for overall water splitting in alkaline.

Electrocatalysts	Cell voltage (V@10 mA· cm^{-2})	Electrolyte	References
**Ir@NiFe-MOF/NF**	**1.47**	**1 M KOH**	**This work**
Ir@Ni-NDC	1.46	1 M KOH	*Angew. Chem. Int. Ed.* **2023**, *62*, e202302220.
Ir-NR/C	1.57	1 M KOH	*Natl. Sci. Rev.* **2020**, *7*, 1340.
IrO_2/V_2O_5	1.49	1 M KOH	*Adv. Sci.* **2022**, *9*, 2104636.
IrO_2-RuO_2/C	1.52	1 M KOH	*Energy Fuels* **2022**, *36*, 1015.
Ru/Co-CAT/CC	1.62 (50 mA· cm^{-2})	1 M KOH	*Adv. Energy Mater.* **2023**, *13*, 2204177.
Pt@LDH-4h	1.49	1 M KOH	*Small* **2023**, *19*, 2207044.
Ir-NSs	1.575	1 M KOH	*Natl. Sci. Rev.* **2020**, *7*, 1340.
NiFe-MOF/NF	1.55	0.1 M KOH	*Nat. Commun.* **2017**, *8*, 15341.
Fe−Co−Ni MOF	1.60	1 M KOH	*J. Am. Chem. Soc.* **2022**, *144*, 3411.

References

[1] G. Kresse, J. Furthmüller, *Comput. Mater. Sci.* **1996**, *6*, 15-50.

[2] G. Kresse, J. Furthmüller, *Phys. Rev. B: Condens. Matter Mater. Phys.* **1996**, *54*, 11169-11186.

[3] P. E. Blöchl, *Phys. Rev. B: Condens. Matter Mater. Phys.* **1994**, *50*, 17953-17979.

[4] G. Kresse, D. Joubert, *Phys. Rev. B* **1999**, *59*, 1758-1775.

[5] J. P. Perdew, J. A. Chevary, S. H. Vosko, K. A. Jackson, M. R. Pederson, D. J. Singh, C. Fiolhais, *Phys. Rev. B: Condens. Matter Mater. Phys.* **1992**, *46*, 6671-6687.

[6] J. P. Perdew, K. Burke, M. Ernzerhof, *Phys. Rev. Lett.* **1996**, *77*, 3865-3868.

[7] S. Grimme, S. Ehrlich, L. Goerigk, *J. Comput. Chem.* **2011**, *32*, 1456-1465.

[8] G. Henkelman, A. Arnaldsson, H. Jónsson, *Comp. Mater. Sci.* **2006**, *36,* 354-360.

[9] E. Sanville, S. D. Kenny, R. Smith, G. Henkelman, *J. Comput. Chem.* **2007**, *28*, 899-908.

[10] A. A. Peterson, F. Abild-Pedersen, F. Studt, J. Rossmeisl, J. K. Nørskov, *Energy Environ. Sci.* **2010**, *3,* 1311-1315.
